# Pien-Tze-Huang alleviates lithocholic acid-induced cholestasis in mice by shaping bile acid-submetabolome

**DOI:** 10.1186/s13020-025-01161-7

**Published:** 2025-07-03

**Authors:** Yan Cao, Yanhong Zhai, Qihong Deng, Shufen Song, Wei Li, Youran Li, Yifan Lu, Jun Li, Zheng Cao, Yuelin Song

**Affiliations:** 1 Department of Laboratory Medicine, Beijing Obstetrics and Gynecology Hospital, Capital Medical University. Beijing Maternal and Child Health Care Hospital, Beijing, 100026 China; 2 Modern Research Center for Traditional Chinese Medicine, Beijing Research Institute of Chinese Medicine, Beijing University of Chinese Medicine, Beijing, 100029 China; 3 Fujian Pien Tze Huang Enterprise Key Laboratory of Natural Medicine Research and Development, Zhangzhou Pien Tze Huang Pharmaceutical Co., Ltd, Zhangzhou, 363000 China

**Keywords:** Pien-Tze-Huang, LCA-induced cholestasis, Bile acid-submetabolome, Widely targeted metabolomics, Therapeutic mechanisms

## Abstract

**Background:**

Cholestasis is one of the most common and devastating manifestations of liver diseases. Although bile acid (BA) metabolism disturbances have been disclosed to be related to the etiopathogenesis of cholestasis, further research is desired to obtain an in-depth understanding of cholestasis. Additionally, only a limited number of treatment approaches are available for this disorder. Pien-Tze-Huang (PTH), a traditional Chinese medicine prescription, has been extensively utilized to treat various liver diseases. However, the effects of PTH on BA-submetabolome and the underlying mechanisms haven’t been revealed.

**Methods:**

A strategy integrating widely targeted metabolomics, untargeted proteomics, and 16S rDNA sequencing, was employed to explore the regulatory effect and the mechanisms of PTH on BA-submetabolome of lithocholic acid (LCA)-induced cholestasis mice. Furthermore, LCA-induced injury HepG2 cells were deployed for efficacy justification and the mechanism exploration.

**Results:**

Both in vivo and in vitro assays demonstrated that PTH could protect liver against LCA-induced injury. Based on the quantitative BA-submetabolome migration and cell viability assays, 3-dehydroCA, CDCA, CA-7-S, HDCA, 3-ketocholanic acid, 7-ketoLCA, and 7,12-diketoLCA were identified as the key BA species correlating with hepatoprotective effects of PTH. Moreover, PTH restored the dramatically deflected BA-submetabolome in cholestasis mice through two different ways. On the one hand, the significantly decreased BA species can be directly supplemented during PTH administration or repaired via upregulating BA-related enzymes. On the other hand, the significantly increased BAs, such as T-*β*-MCA, TCDCA, TCA, TLCA, TMDCA, TUDCA, and TDCA, should be eliminated by the increased abundance of *Lactobacillaceae* and *Lactobacillus*.

**Conclusions:**

PTH alleviates cholestasis by synergistically regulating certain BA species, enzymes and gut microbiota, leading to holistic BA-submetabolome shaping.

**Supplementary Information:**

The online version contains supplementary material available at 10.1186/s13020-025-01161-7.

## Introduction

Bile acids (BAs), a large group of structurally related steroids, serve as the primary components of vertebrate bile. In hepatocytes, cholesterol is metabolized to primary BAs by a series of enzymes, such as CYPs 7A1, 8B1, and 27A1, and subsequently conjugated with taurine or glycine before biliary secretion. When being modified by intestinal microbiota (*e.g., bifidobacteria* and *lactobacilli*), BAs usually undergo deconjugation, epimerization, oxidation, and dehydroxylation to produce secondary BAs, which are subsequently delivered to the liver via diverse transporters, *e.g.,* OATP and BCRP. When BAs connect to receptors such as farnesoid X receptor (FXR) and Takeda G protein-coupled receptor 5 (TGR5), they also function as signalling molecules within this network to trigger downstream signalling pathways [1]. Consequently, all members within the metabolism network work together to regulate the size and composition of BA pool, also called BA-submetabolome and these quantitative characteristics are essential for the maintenance of BA homeostasis [2, 3].

Cholestasis is characterized by the intrahepatic accumulation of potentially toxic BAs resulted from disturbed biosynthesis, metabolism, or transport [4]. Various liver diseases, including biliary atresia, primary biliary cholangitis (PBC), and primary sclerosing cholangitis (PSC), typically accompany with this clinical appearance [5, 6]. Although the elevated total bile acid (TBA) value is commonly implemented for the cholestasis diagnosis in clinical practices, disorders of the BA metabolism network during cholestasis can also manifest as altered primary BA to secondary BA ratios and altered conjugated BA to unconjugated BA ratios in the serum [7]. Recently, the gut microbiota‒bile acid axis has become a research hotspot and has been widely used in the study of the pathogenesis of cholestasis [8]. Overall, the mechanism clarification for the cross-talk among host metabolism, the gut microbiota and BA-submetabolome is essential for elucidating the pathological mechanism of cholestasis. Furthermore, although ursodeoxycholic acid (UDCA) is an FDA-approved medicine for the treatment of cholestasis, it usually suffers from unsatisfactory benefits [9].

Pien-Tze-Huang (PTH), a famous traditional Chinese medicine prescription, is primarily composed of natural musk, natural bezoars, snake bile, and notoginseng, and has been utilized to treat various liver inflammation and tumor for centuries [10]. The anti-inflammatory and immunoregulatory properties of PTH have been the primary topics of current study on the therapeutic outcomes [11–13]. Importantly, natural bezoars and snake bile, which are classified as medicinal bile, possess significant potential to ameliorate hepatobiliary diseases due to their high concentration of various BAs [14]. However, from the perspective of BA metabolism, there are several challenges towards clarifying the active compounds and the related therapeutic mechanisms for liver disorders. In our previous report [15], the administration of a single BA species initiated serial follow-up reactions within the BA metabolism network, leading to temporal fluctuations of the entire BA-submetabolome. Moreover, PTH is a formula rich in BAs that closely resemble those produced endogenously, and even by isotope labelling, it is extremely difficult to determine the sequence of the chained reaction after numerous BAs entering the body and to distinguish between functional BAs, corresponding to those directly complementing endogenous BAs, and altered BAs by PTH. Although several individual BAs have been identified as key signalling molecules, different BA species might show distinct signalling properties [16]. Representatively, LCA and deoxycholic acid (DCA) are the preferred ligands for Takeda G protein-coupled receptor 5 (TGR5), whereas cholic acid (CA) and chenodeoxycholic acid (CDCA) are the most potent ligands for farnesoid X receptor (FXR) [17]. In other words, the impact of the biological outcomes of PTH should be holistically resulted from the action of different components and their synergistic or antagonistic activities.

Therefore, efforts were made here to clarify the alterations in BA metabolism network reshaped by PTH in cholestasis mice in terms of participants, such as BA-submetabolome, BA-related enzymes, and intestinal flora. The findings obtained in the current study are envisioned: (1) to assess the effectiveness of quantitative BA-submetabolome to reflect both pathological and pharmacological statuses; (2) to explore functional BAs owning direct hepatoprotective effects on PTH; (3) to reveal BAs that can be directly supplemented after PTH administration and indirectly reshaped by functional BAs in PTH through regulating the expression of metabolic enzymes or the composition of the intestinal flora; and (4) to provide an eligible strategy for the investigation of medicinal substances and the action mechanisms of traditional medicines being rich of endogenous BAs.

## Materials and methods

### Chemicals, reagents and assay kits

Pien-Tze-Huang (PTH) troches were supplied by Zhangzhou Pien-Tze-Huang Pharmaceutical Co., Ltd. An in-house BA library [18] containing as many as 120 items was used for compound identification. LCA and UDCA, as well as astragaloside IV that served as the internal standard (IS), were purchased from Sigma-Aldrich (Merck, Darmstadt, Germany). The purity of each authentic compound was determined to be greater than 98% by LC–IT-TOF–MS (Shimadzu, Tokyo, Japan). Ultrafiltration units with 10 kDa molecular weight cut-off (MWCO) were purchased from Sartorius (Goettingen, Germany). Minimum essential medium (MEM) and trypsin (0.25% with EDTA) were commercially obtained from Gibco (Thermo-Fisher, Pittsburgh, PA). All cell culture reagents were purchased from Gibco, and a cell counting kit-8 (CCK-8) kit was supplied by Beijing Lambolid Co., Ltd. (Beijing, China).

LC–MS grade methanol, acetonitrile (ACN), ammonium formate, and formic acid were commercially obtained from Thermo-Fisher. DMSO was supplied by Merck (Darmstadt, Germany). Deionized water was prepared in-house on a Milli-Q Integral water purification system (Millipore, Bedford, MA).

### Animal experiments

All animal experiment protocols were approved by the Animal Ethics Committee of Beijing University of Chinese Medicine (Beijing, China). The detailed flowchart is shown in Figure S1. Briefly, 48 male 8-week-old C57BL/6 J mice were maintained for 1 week for adaptive feeding and then randomly divided into six groups (*n* = 8 for each), such as the control group, the model group (LCA-induced cholestasis, 150 mg·kg^−1^), UDCA group (positive control group, 150 mg·kg^−1^), the low dosage of PTH (PTH-L, 75 mg·kg^−1^), the middle dosage of PTH (PTH-M, 150 mg·kg^−1^) and the high dosage of PTH (PTH-H, 300 mg·kg^−1^). Drug dosage for animal experiments was calculated and adjusted for body weight based on manufacturer's recommended dosage for adults. Afterwards, different dosing regimens were assigned to each group. At the end point of this flowchart, the mice were euthanized, and the blood was sampled from each animal and divided into two portions, including one for measurement of hepatic injury indices such as serum alanine aminotransferase (ALT), aspartate transaminase (AST), alkaline phosphatase (ALP), TBA, and total bilirubin (TBIL) levels, and the other for quantitative BA-submetabolome characterization. Following perfusion, the liver was immediately removed from each mouse and divided into two portions, including one for hematoxylin–eosin (HE) staining and the other for quantitative BA-submetabolome characterization. The intestine was sampled, cut into duodenum, jejunum, and ileum portions, and stored at − 80 °C until usage.

### Quantitative BA-submetabolome characterization

Serum, liver, and ileal tissues were homogenized within each animal for quantitative BA-submetabolome characterization via applying a well-defined analytical program [18]. LC–MS/MS measurements were accomplished by a LC-20AD_XR_ LC (Shimadzu, Kyoto, Japan) coupled with a Qtrap5500 MS/MS instrument (SCIEX, Foster City, CA) equipping an electrospray ionization (ESI) interface. Chromatographic separations were performed on a Waters Acquity UPLC HSS T3 column (2.1 × 100 mm, 1.8 μm, Milford, MA, USA). Moreover, Quality control (QC) samples were measured after every 10 injections to evaluate the method stability. The details of sample preparation along with data collection and processing are illustrated in Supplemental Information.

### In vitro cell assays

Cell viability was assayed by CCK-8 method. HepG2 cells were seeded at a density of 5000 cells/well in 96-well plates for 24 h and subsequently incubated with 10 μL/well CCK-8 solution for another two hours at 37 °C. The optical density value was measured at 450 nm using Multiskan GO Microplate Reader (Thermo Scientific, Rockford, IL, USA). The cell viability was calculated by the equation as follows: Cell viability (%) = (cell viability of drug group–cell viability of model group)/(cell viability of control group–cell viability of model group) × 100.

### Untargeted proteomics measurements

Liver S9 fractions were digested by following the partial filter-aided sample preparation (FASP) method [19] and analysed by the Ultimate3000 nano RSLC system (Thermo Scientific) coupling to an LTQ Orbitrap Velos device. The separations of the tryptic peptide mixtures were conducted on an EASY-Spray^™^ column (0.075 × 100 mm, 3 μm, 100 Å; Thermo-Fisher Scientific) by programming a linear increment for the mobile phase consisting of ACN (solvent B) and 0.1% aqueous formic acid (solvent A) from 5%B to 100%B at a total flow rate of 300 nL·min^−1^ over 90 min.

### 16S rDNA sequencing of the gut microbiota

The microbiome of the ileum was measured by high-throughput sequencing techniques. Details on data processing can be found in the Supplementary Materials.

### Statistical analysis

Statistical analysis was performed using GraphPad Prism 8.0 software (San Diego, CA, USA), and each data was expressed as the mean ± SD. SIMCA-P software (Version 14.1, Umetrics, Umeå, Sweden) was deployed for multivariate statistical analysis. Statistical differences were determined using a one-way ANOVA followed by Tukey’s post hoc test for multiple comparisons, and *P* < 0.05 was considered to be statistically significant difference.

## Results

### Therapeutic outcomes of PTH towards LCA-induced cholestatic liver injury mice

**Fig. 1:**
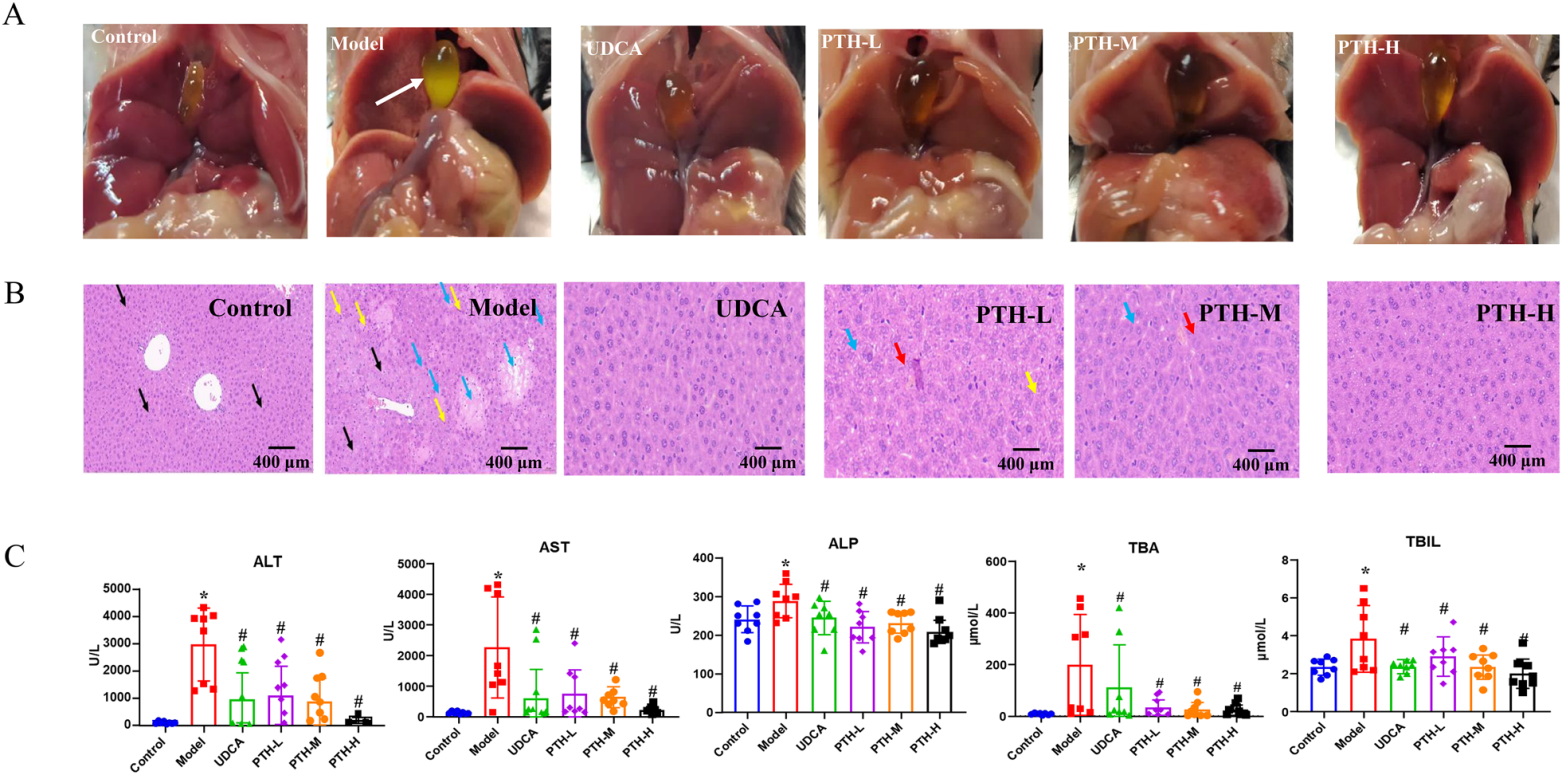
Hepatoprotective effect of PTH against LCA‐induced cholestatic liver damage in C57BL/6 J mice. **A** Morphology of representative liver and gallbladder samples from each group. **B** Representative H&E-stained liver sections (× 20). Areas of severe liver necrosis are indicated with arrows (black arrow: microvesicular steatosis; blue arrow: foci of hepatocellular necrosis; yellow arrow: inflammatory cell infiltration; red arrow: hyperplasia of bile duct cells). **C** Serum levels of ALT, AST, TBIL, ALP, and TBA (*P < 0.05, compared with the control group; ^#^*P* < 0.05, compared with the model group)

As a secondary BA species, LCA is hepatotoxic and plays an important role in liver injury [20]. After 4 days of LCA treatment, the results of both morphological and histological assays revealed severe liver necrosis, diffuse vacuolization, infiltrating neutrophils, and gallbladder enlargement for the model group. Importantly, the liver necrosis degree offered by histological assay revealed that pretreatment with UDCA or PTH significantly suppressed these pathological alterations (Fig. 1A, B). Being consistent with the histopathologic results, LCA administration dramatically elevated the serum ALT, AST, ALP, TBIL, and TBA levels, corresponding to 43.9-, 16.3-, 1.2-, 1.6-, and 21.7-fold greater than those in the control group, respectively. Exactly, PTH treatment reversed these LCA‐induced increments with a dose-dependent manner (Fig. 1C). Consequently, these results demonstrated that PTH provides dose-dependent protection against LCA‐induced intrahepatic cholestasis and hepatotoxicity.

### Quantitative BA-submetabolome characterization

**Fig. 2:**
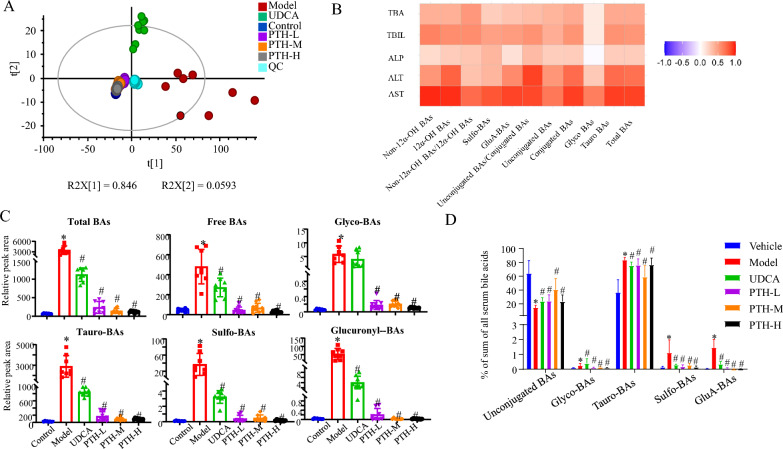
Serum BA analysis. **A** PCA score plots derived from the serum bile acid levels of the control, model, UDCA, PTH-L, PTH-M and PTH-H groups. **B** Correlation heatmap (Pearson correlation) of bile acid patterns and biochemical indicators. **C** Variations in different types of bile acids. **D** The ratios of different types of bile acids to total serum bile acids (*P < 0.05, compared with the control group; ^#^*P* < 0.05, compared with the model group)

Under pathological and physiological statuses, BA-submetabolome usually exhibits significant migration. Several studies have demonstrated that tighter correlations occur for BA-submetabolome with liver disease progression than the absolute level of total or certain individual BA species; hence, quantitative BA-submetabolome characterization has been performed in serum by applying the widely targeted metabolomics strategy [21–23]. Principal component analysis (PCA), a tool for reducing high-dimensional data into a lower-dimensional space, was employed for preliminary data processing. As shown in Fig. 2A, the samples from different groups are separated; however, the dots are well clustered within each group. QC samples gathered tightly around the core of all samples, indicating steady and reliable LC–MS system performance throughout the measurement queue. Moreover, the results of repeatability, stability, and intra- and inter-day variations indicated that this robust analytical method was applicable in BA-submetabolome measurements (Figure S2 and Table S1). The samples belonging to the model group were clearly separated from the control group (Fig. 2A), suggesting that cholestasis caused significant BA-submetabolome disturbance. Compared with the model group, the scattering style of samples from UDCA group were similar to the control group, and samples from PTH-L, PTH-M, and PTH-H groups exhibited tighter assembly than those of the control group, suggesting that the levels of some metabolites recovered after the oral administration of UDCA or PTH. Moreover, Spearman test was performed, and positive correlations were observed between BA patterns and biochemical indicators, indicating that excessive accumulation of intrahepatic BAs may result in liver injury, suggesting quantitative BA-submetabolome characterization to be meaningful for cholestasis diagnosis and drug efficacy evaluation (Fig. 2B). Notably, unconjugated/conjugated BA ratios showed the great correlation with AST and ALT. Moreover, all the levels of TSBA, unconjugated BAs, tauro-BAs, glycol-BAs, and glucuronyl- and sulfo-conjugated BAs in the model group were significantly higher than those in the control group, and these variations could be reversed with UDCA or PTH treatment (Fig. 2C). In contrast to that in the control group, the ratio of unconjugated BAs to TSBA was significantly lower in the model group (Fig. 2D). Therefore, the biotransformation between unconjugated BAs and conjugated BAs played a key role for cholestasis.

**Fig. 3:**
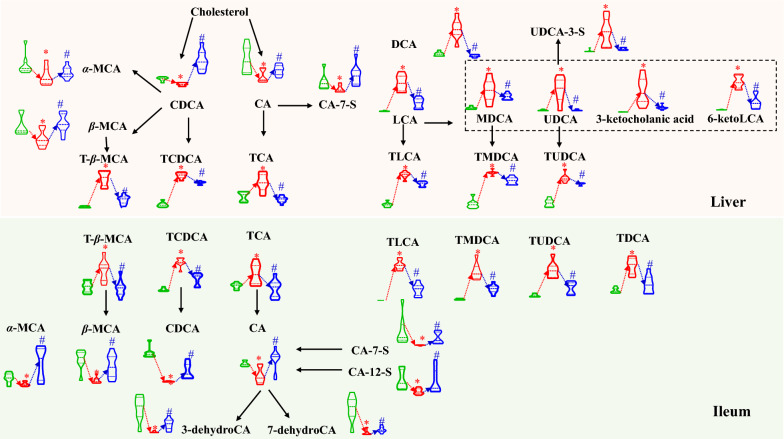
Significantly altered BA species in the liver and ileum between the control, model, and PTH-H groups. (*P < 0.05, compared with the control group; ^#^*P* < 0.05, compared with the model group. Green: control group; red: model group; blue: PTH-H group

**Fig. 4:**
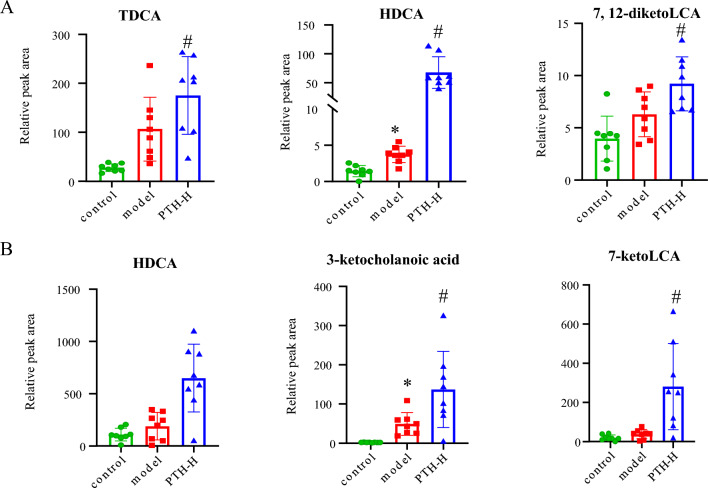
BAs in the liver (**A**) and ileum (**B**) that significantly increased in PTH-H group (in comparison to those in the model group) and nonetheless, insignificantly decrease in the model group (in comparison to those in the control group). (*P < 0.05, compared with the control group; ^#^*P* < 0.05, compared with the model group.)

Because BAs are biosynthesized in the liver and metabolized by gut bacteria in the intestine, the BA-submetabolome of the liver and ileum was also quantitatively measured. Following PCA treatment, the loading plots of the control group vs. the model group and the model group vs. the PTH-H group are shown in Figures S3 and S4, respectively, and the variations in outliers with statistically significant differences were compared among the three groups (Fig. 3). Primary BAs such as CA, CDCA, *α*-muricholic acid (*α*-MCA), and *β*-muricholic acid (*β*-MCA) were significantly decreased in the liver samples of the model group compared with those belonging to the control group. However, the levels of taurine-conjugated species, such as tauro-*β*-muricholic acid (T-*β*-MCA), taurochenodeoxycholic acid (TCDCA), and taurocholic acid (TCA), dramatically grew. In addition, LCA treatment increased the levels of certain secondary BA species, including DCA and LCA. Because murideoxycholic acid (MDCA), UDCA, 3-ketocholanic acid, and 6-ketoLCA are LCA metabolites, the administration of LCA also resulted in an extensive increment for these species as well as their taurine-conjugated products, such as TLCA, TMDCA, and TUDCA. Similarly, taurine-conjugated BAs such as T-*β*-MCA, TCDCA, TCA, TLCA, TMDCA, TUDCA, and TDCA were elevated in the ileum. Meanwhile, decrements occurred for CA, CDCA, *α*-MCA, *β*-MCA, along with other CA metabolites, such cholic acid-7-sulfate (CA-7-S), cholic acid-12-sulfate (CA-12-S), 3-dehydroCA, and 7-dehydroCA. Importantly, PTH treatment significantly reversed these interrupted BAs (Fig. 3). In other words, these substantially reversed BAs are endogenous BAs that were reshaped by PTH during cholestasis. Furthermore, several BA species, including TDCA, HDCA, and 7,12-diketoLCA in the liver and HDCA, 3-ketocholanic acid, and 7-ketoLCA in the ileum, significantly increased in PTH-H group but did not significantly decrease in the model group (Fig. 4). These species might serve as functional BAs in PTH-H.

### Effects of potential functional BAs on LCA-induced hepatocellular injury in vitro

**Fig. 5:**
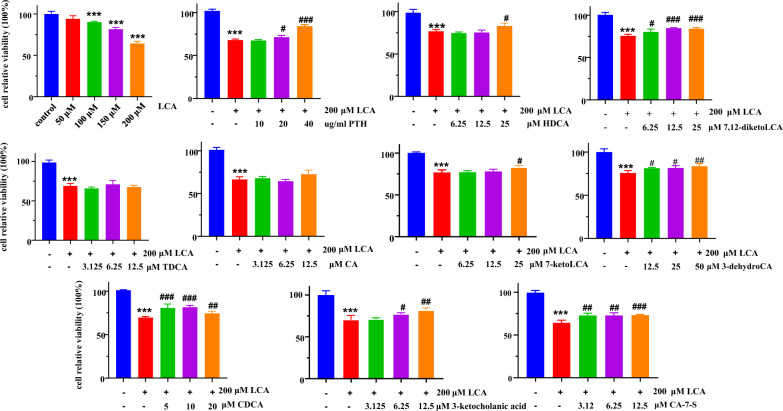
Effects of PTH and potential functional BAs on cell viability. (***P < 0.001, compared with the control group; ^#^*P* < 0.05, ^##^*P* < 0.01, and ^###^*P* < 0.001, compared with the model group.)

The significantly elevated BAs in the PTH-H group compared to those in the model group were considered as potential functional BAs, such as CA, CDCA, 3-dehydro CA, 7-dehydro CA, CA-7-S, CA-12-S, TDCA, HDCA, 7,12-diketoLCA, HDCA, 3-ketocholanic acid, and 7-ketoLCA. To investigate the protective effects of PTH and potential functional BAs on HepG2 cell injury, we constructed an LCA-induced hepatocellular injury model. HepG2 cells were treated with progressive concentrations of LCA (50–200 μM) for 24 h, and the viability of HepG2 cells was assayed with CCK-8 method (Fig. 5). As a result, LCA decreased cell viability in a dose-dependent manner, and particularly, cell survival rate of approximately 65% when the concentration was increased to 200 μM. Hence, 200 μM was selected to establish HepG2 cell injury model. Moreover, HepG2 cells were exposed to different concentration levels of PTH, CA, CDCA, CA-7-S, HDCA, TDCA, 3-ketocholanic acid, 7-ketoLCA, and 7,12-diketoLCA to evaluate their hepatocellular protective outcomes. As shown in Fig. 5, PTH, 3-dehydroCA, CDCA, CA-7-S, HDCA, 3-ketocholanic acid, 7-ketoLCA, and 7,12-diketoLCA exhibit notable protective effects against LCA-induced hepatocellular injury, particularly at high concentration levels. Although TDCA and CA slightly improved cell viability, there was no significant effect on cell viability at the dose we used.

### Effects of PTH treatment on hepatic BA–related protein expression in mice with cholestasis

**Fig. 6:**
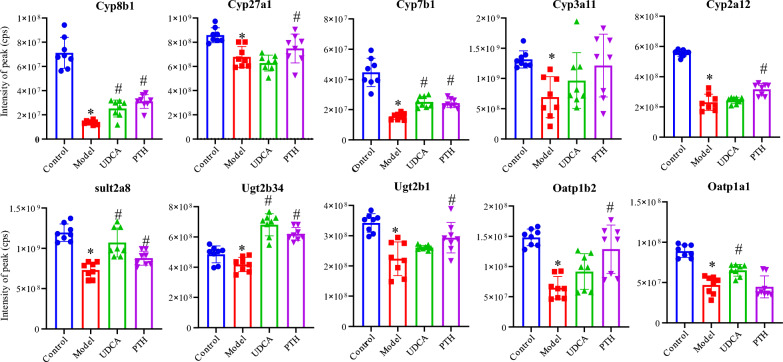
Expression of bile acid–related proteins in the liver. (*P < 0.05, compared with the control group; ^#^*P* < 0.05, compared with the model group.)

As aforementioned, PTH restored the altered BA profiles in LCA-induced cholestatic liver injury. To investigate the underlying therapeutic mechanisms of PTH, we performed nontargeted proteomics analysis for liver samples. Because the phase I metabolic enzyme cytochrome P450s (CYP450s), phase II metabolic enzymes such as uridine diphospho-glucuronosyl transferases (UGTs), and sulfo-transferases (SULTs) are critical participants in biosynthesis pathways, a total of 38 proteins correlating to CYP450s, SULTs, and UGTs were selected (Table S2). Enzymes involved in the classical pathway of BA synthesis, such as CYPs 7A1, 8B1, and 27A1, were significantly downregulated in the model group. However, in the groups that received PTH or UDCA treatment, their levels returned to normal levels (Fig. 6). Additionally, a number of enzymes were markedly downregulated in the model group but upregulated in UDCA or PTH group, and noteworthily, PTH exhibited the greater effect. These enzymes included those involved in BA hydroxylation (CYPs 3A11 and 2A12), BA sulfation (SULT 2A8), and BA glucuronidation (UGTs 2B34 and 2B1). Furthermore, OATPs 1A1 and 1B2, two transporters that mediate bile salts from the blood to the bile, were significantly decreased in the model group but returned following UDCA or PTH administration.

### Effects of PTH treatment on BA–related gut microbiota abundance in mice with cholestasis

**Fig. 7:**
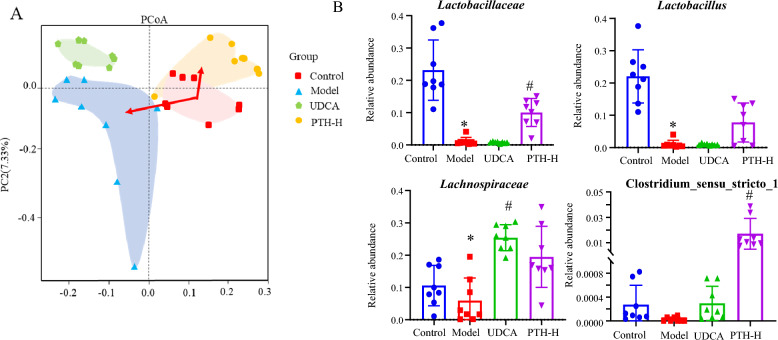
PCoA analysis (**A**) and the relative abundance of bile acid–related gut microbiota in the four groups (**B**) (*P < 0.05, compared with the control group; ^#^*P* < 0.05, compared with the model group)

Because the gut microbiota plays a significant role in shaping the structure and composition of BAs, 16S rDNA sequencing was performed for cholestasis mice to explore the effect of PTH treatment on BA-related gut microbiota composition. Bacterial* α* diversity was evaluated using Shannon, Simpson, ACE, and Chao indices, whereas *β* diversity was assessed by principal coordinate analysis (PCoA). Compared with the control group, a lower *α* diversity in the gut microbiota was observed in the model group, but the difference was not statistically significant. PTH administration improved the diversity of the gut microbiota in cholestasis mice, and the performance was also supported by the considerably higher Shannon and Simpson indices than the model group (Figure S5). With a smaller distance between the control and PTH groups than the model group against PTH group, PCoA revealed notable variations for the gut microbiota within each group (Fig. 7A).

The *Lactobacillaceae* family has strong bile salt hydrolases that may deconjugate taurine- or glycine-conjugated BAs into unconjugated BAs [24, 25]. Compared with the control group, the model group presented significant decreases in *Lactobacillus* and *Lactobacillaceae* abundances, whereas compared with the model group, PTH-H group presented substantial increases in regards of both *Lactobacillus* and *Lactobacillaceae* abundances (Fig. 7B). Additionally, as a mediator of the conversion of primary BAs to secondary BAs, *Lachnospiraceae* was decreased significantly in the model group. *Lachnospiraceae* abundance increased with PTH treatment, although this increase was not statistically significant. Compared with the model group, the abundance of *Lachnospiraceae* in UDCA group was markedly greater. *Clostridium*, which mediates 7*α*-dehydroxylation activity [26], was significantly lower in the model group than in the control group, while a significant increase was detected in PTH-H group.

## Discussions

PTH has been used for the treatment of various types of liver inflammation and tumor for more than ten decades, and the present study revealed that PTH protects against LCA-induced cholestatic liver injury, as evidenced by improved liver morphology and histology, as well as a significant decrease in the serum ALT/AST/ALP and serum total BAs. Notably, significant correlations between BA patterns and biochemical indicators indicate the reliability of BA profiling in characterizing disease status and pharmacological treatment efficacy.

BA-submetabolome can be shaped by a variety of regulatory factors, including metabolic enzymes, transporters, and the gut microbiota. Moreover, BAs also serve as signalling molecules for these regulatory variables. Thus, determining the causal linkages between the multiple disorders that emerge in the BA metabolic network during cholestasis is challenging. As previously reported [27, 28], suddenly increased hydrophobic BAs such as LCA and DCA in cholestasis cause toxic effects, leading to “upstream” bile duct and liver parenchymal damage. Additionally, the negative feedback regulatory mechanism of the FXR pathway prevents the production of primary BAs due to increased BAs. This finding was supported in this study by the noticeably decreased CA, CDCA, *α*-MCA, and *β*-MCA in the model group. However, their taurine-conjugated BAs increased dramatically, possibly because of a reduced number of *Lactobacillaceae* and *Lactobacillus bacteria*, which deconjugate glycine- or taurine-conjugated BAs into unconjugated BAs [24, 25]. Therefore, restoring the gut microbiota and restoring BA homeostasis may cooperate to protect against cholestasis.

Notably, BA-submetabolome in PTH are similar to those in humans; therefore, PTH can improve cholestasis in two ways. First, BAs in PTH can directly replenish the reduced BAs in mice with cholestasis when they are administered. Second, functional BAs in PTH can indirectly regulate disrupted BAs in mice with cholestasis by regulating enzymes or the gut microbiota. However, because PTH and human BAs are remakably similar, distinguishing between functional BAs derived from PTH and reshaped endogenous BAs is difficult [29]. Compared with those in the control group, CA, CDCA, 3-dehydro CA, 7-dehydro CA, CA-7-S, CA-12-S, *α*-MCA, and *β*-MCA were significantly reduced in the model group but significantly greater after PTH treatment. In accordance with our previous research [30], CA, CDCA, 3-dehydro CA, 7-dehydro CA, CA-7-S, and CA-12-S were found in snake bile. Furthermore, the oxidation metabolites of CA, such as 3-dehydro CA and 7-dehydro CA, as well as the hydroxylation metabolites of CDCA, such as *α*-MCA and *β*-MCA, are further facilitated by the upregulation of Cyp3a11 and Cyp2a12. Additionally, the elevated levels of CA-7-S and CA-12-S were further supported by the upregulation of Sult2a8. Therefore, in mice with cholestasis, PTH can regulate the levels of CA, CDCA, 3-dehydro CA, 7-dehydro CA, CA-7-S, and CA-12-S in two ways, including directly through supplementation and indirectly through regulation.

However, several BAs can be indirectly regulated only by PTH via metabolic enzymes, transporters, or the gut microbiota. For example, *α*-MCA and *β*-MCA are rodent specific and significantly increased after PTH administration [31]. And those BAs significantly increased in the model group but then reversed after PTH supplementation, such as T-*β*-MCA, TCDCA, TCA, TLCA, TMDCA, TUDCA, and TDCA. The metabolic pathways of taurine-conjugated BAs primarily involve conjugation mediated by BAAT and BACS in the liver, as well as deconjugation mediated by the intestinal flora in the intestine [32]. Unfortunately, BAAT and BACS were not detected in the untargeted proteomics; nevertheless, the increased abundance of *Lactobacilli* indicated that PTH promoted the deconjugation of taurine-conjugated BAs in the intestine. Furthermore, the reduction of LCA, DCA, MDCA, UDCA, 3-ketocholanic acid, and 6-ketoLCA may result from PTH inhibiting the absorption of LCA.

FXR and TGR5 are essential bile acid-activated receptors that regulate BA, glucose, and lipid metabolism. Compared with those in the model group, the significantly elevated BAs in the PTH-H group can be considered as potential functional BAs. Further in vitro investigations revealed that HDCA, 7,12-diketoLCA, 7-ketoLCA, 3-dehydroCA, CDCA, 3-ketocholanic acid, and CA-7-S have protective effects on HepG2 cells exposed to LCA-induced injury. These BAs can be regarded as functional BAs in PTH that ameliorate cholestasis. CDCA is an endogenous FXR agonist, with FXR regulating homeostasis and exerting cytoprotective effects [33]. It upregulates NLRP3 in liver macrophages through FXR receptors, enhancing intestinal permeability and modulating microbiota to alleviate liver injury [34]. Recent work further demonstrates CDCA mediated modulation of the cholestatic niche through FXR/Myc/P-selectin axis in liver endothelial cells [35]. Although HDCA inhibits FXR in the intestine, it can advance lipid catabolism through fatty acid‒hepatic peroxisome proliferator-activated receptor alpha (PPARα) signalling, which in turn upregulates hepatic FXR. By upregulating Cyp8b1 expression, HDCA can also activate the alternate pathway of bile acid biosynthesis [36]. Notably, both HDCA and CA-7-S improve glucose homeostasis and hepatic metabolism by stimulating TGR5/FXR-dependent GLP-1 secretion [37–39].

Nevertheless, this study has several limitations that need to be acknowledged. First, although the alterations in BA sub-metabolome were characterized, key metabolic enzymes (e.g., BAAT, BACS) and transporters (e.g., ASBT, MRP2) were not quantified, precluding reconstruction of the comprehensive BA metabolic network in PTH-treated cholestatic mice. Future studies should employ targeted proteomics to resolve this. Second, the origins of functional BAs remain unclear, whether they derive directly from PTH or its in vivo metabolites. Most critically, their activation mechanisms for FXR/TGR5 receptors require elucidation. Therefore, follow-up work will prioritize the BA-FXR/TGR5-GLP-1 pathway to define the roles of CA-7-S and other key BAs identified in PTH. The protective effects of *α*-MCA, *β*-MCA, 7,12-diketoLCA, 7-ketoLCA, 3-dehydroCA, 3-ketocholanic acid, and CA-12-S on hepatocytes and their regulatory roles in BA receptors warrant further investigation.

## Conclusion

Although numerous attempts have been made to reveal the mechanism being responsible for that PTH ameliorates liver disease, very few studies have investigated the functional BAs associated with PTH and their regulatory effects on BA metabolic network. In current study, quantitative BA-submetabolome was applied to evaluate the pathological status of cholestasis as well as the pharmaceutical effects of PTH. Moreover, functional BAs in PTH and the reshaped BA-submetabolome in mice with cholestasis were identified according to the variations in BA-submetabolome within different groups. Furthermore, the findings of untargeted proteomics combined with 16S rDNA sequencing revealed that these functional BAs can reshape BA metabolomic network in direct and indirect ways. More importantly, this research provides meaningful insights into the pharmacological substances and mechanisms of action of TCMs containing endogenous components.

## Supplementary Information

Additional file 1

## Data Availability

All data for the duration of the study can be found in this article and appendix. And the raw sequencing data was deposited in the public repository–NCBI Gene Expression Omnibus (GEO). The sequencing dataset are available through the NCBI Gene Expression Omnibus (GEO) under accession number SUB15269776: https://submit.ncbi.nlm.nih.gov/subs/sra/.

## Abbreviations

ACN: Acetonitrile
ALP: Alkaline phosphatase
ALT: Alanine aminotransferase
ASBT: Apical sodium-dependent bile acid transporter
AST: Aspartate transaminase
BA: Bile acid
CA: Cholic acid
CA-7-S: Cholic acid-7-sulfate
CA-12-S: Cholic acid-12-sulfate
CCK-8: Cell counting kit-8
CDCA: Chenodeoxycholic acid
CYP450: Cytochrome P450
DCA: Deoxycholic acid
ESI: Electrospray ionization
FASP: Filter-aided sample preparation
FXR: Farnesoid X receptor
GLP-1: Glucagon-like peptide-1
HE: Hematoxylin‒eosin
LCA: Lithocholic acid
*α*-MCA: *α*-Muricholic acid
*β*-MCA: *β*-Muricholic acid
MDCA: Murideoxycholic acid
MEM: Minimum essential medium
MRP2: Multidrug Resistance-associated Protein 2
PBC: Primary biliary cholangitis
PCA: Principal component analysis
PCoA: Principal coordinate analysis
PSC: Primary sclerosing cholangitis
PTH: Pien-Tze-Huang
SULT: Sulfo-transferase
TBA: Total bile acid
TBIL: Total bilirubin
TGR5: Takeda G protein-coupled receptor 5
UDCA: Ursodeoxycholic acid
UGT: Uridine diphospho-glucuronosyl transferase

## References

[1] Fiorucci S, Distrutti E. Bile acid-activated receptors, intestinal microbiota, and the treatment of metabolic disorders. Trends Mol Med. 2015;21(11):702–14. 26481828 10.1016/j.molmed.2015.09.001

[2] Sinal CJ, Tohkin M, Miyata M, Ward JM, Lambert G, Gonzalez FJ. Targeted disruption of the nuclear receptor FXR/BAR impairs bile acid and lipid homeostasis. Cell. 2000;102(6):731–44. 11030617 10.1016/s0092-8674(00)00062-3

[3] Eloranta JJ, Kullak-Ublick GA. The role of FXR in disorders of bile acid homeostasis. Physiology (Bethesda). 2008;23:286–95. 18927204 10.1152/physiol.00020.2008

[4] Yang K, Kock K, Sedykh A, Tropsha A, Brouwer KL. An updated review on drug-induced cholestasis: mechanisms and investigation of physicochemical properties and pharmacokinetic parameters. J Pharm Sci. 2013;102(9):3037–57. 23653385 10.1002/jps.23584 PMC4369767

[5] Kullak-Ublick GA, Meier PJ. Mechanisms of cholestasis. Clin Liver Dis. 2000;4(2):357–85. 11232196 10.1016/s1089-3261(05)70114-8

[6] Yu L, Liu X, Yuan Z, Li X, Yang H, Yuan Z, et al. SRT1720 alleviates ANIT-induced cholestasis in a mouse model. Front Pharmacol. 2017;8:256. 28553227 10.3389/fphar.2017.00256 PMC5425580

[7] Manzotti C, Casazza G, Stimac T, Nikolova D, Gluud C. Total serum bile acids or serum bile acid profile, or both, for the diagnosis of intrahepatic cholestasis of pregnancy. Cochrane Database Syst Rev. 2019;7(7):CD012546. 31283001 10.1002/14651858.CD012546.pub2 PMC6613619

[8] Yang X, Xu Y, Li J, Ran X, Gu Z, Song L, et al. Bile acid-gut microbiota imbalance in cholestasis and its long-term effect in mice. mSystems. 2024;9(7): e0012724. 38934542 10.1128/msystems.00127-24 PMC11265269

[9] Levy C. Primary biliary cholangitis: treatment options finally expand. Hepatology. 2017;65(4):1405–7. 28093785 10.1002/hep.29053

[10] Zhang Y, Hua L, Lin C, Yuan M, Xu W, Raj DA, et al. Pien-Tze-Huang alleviates CCl(4)-induced liver fibrosis through the inhibition of HSC autophagy and the TGF-beta1/Smad2 pathway. Front Pharmacol. 2022;13: 937484. 36188553 10.3389/fphar.2022.937484 PMC9523731

[11] Lian B, Cai L, Zhang Z, Lin F, Li Z, Zhang XK, et al. The anti-inflammatory effect of Pien Tze Huang in non-alcoholic fatty liver disease. Biomed Pharmacother. 2022;151: 113076. 35550529 10.1016/j.biopha.2022.113076

[12] Zhao R, Zhang Q, Liu W, Lin Y, He Y, Chang D, et al. Pien Tze Huang attenuated acetaminophen-induced liver injury by autophagy mediated-NLRP3 inflammasome inhibition. J Ethnopharmacol. 2023;311: 116285. 36933874 10.1016/j.jep.2023.116285

[13] Zheng H, Wang X, Zhang Y, Chen L, Hua L, Xu W. Pien-Tze-Huang ameliorates hepatic fibrosis via suppressing NF-kappaB pathway and promoting HSC apoptosis. J Ethnopharmacol. 2019;244: 111856. 30959141 10.1016/j.jep.2019.111856

[14] Qiao X, Ye M, Pan DL, Miao WJ, Xiang C, Han J, et al. Differentiation of various traditional Chinese medicines derived from animal bile and gallstone: simultaneous determination of bile acids by liquid chromatography coupled with triple quadrupole mass spectrometry. J Chromatogr A. 2011;1218(1):107–17. 21111425 10.1016/j.chroma.2010.10.116

[15] Cao Y, Li W, Gong X, Niu X, Zheng J, Yu J, et al. Widely quasi-quantitative analysis enables temporal bile acids-targeted metabolomics in rat after oral administration of ursodeoxycholic acid. Anal Chim Acta. 2022;1212: 339885. 35623780 10.1016/j.aca.2022.339885

[16] Lefebvre P, Cariou B, Lien F, Kuipers F, Staels B. Role of bile acids and bile acid receptors in metabolic regulation. Physiol Rev. 2009;89(1):147–91. 19126757 10.1152/physrev.00010.2008

[17] Fiorucci S, Biagioli M, Zampella A, Distrutti E. Bile acids activated receptors regulate innate immunity. Front Immunol. 2018;9:1853. 30150987 10.3389/fimmu.2018.01853 PMC6099188

[18] Cao Y, Li W, Chen W, Niu X, Wu N, Wang Y, et al. Squared energy-resolved mass spectrometry advances quantitative bile acid submetabolome characterization. Anal Chem. 2022;94(44):15395–404. 36286389 10.1021/acs.analchem.2c03269

[19] Coleman O, Henry M, Clynes M, Meleady P. Filter-Aided Sample Preparation (FASP) for improved proteome analysis of recombinant Chinese hamster ovary cells. Methods Mol Biol. 2017;1603:187–94. 28493131 10.1007/978-1-4939-6972-2_12

[20] Nguyen TT, Ung TT, Li S, Sah DK, Park SY, Lian S, et al. Lithocholic Acid Induces miR21, Promoting PTEN Inhibition via STAT3 and ERK-1/2 Signaling in Colorectal Cancer Cells. Int J Mol Sci. 2021;22(19):10209. 34638550 10.3390/ijms221910209 PMC8508661

[21] Sang C, Wang X, Zhou K, Sun T, Bian H, Gao X, et al. Bile acid profiles are distinct among patients with different etiologies of chronic liver disease. J Proteome Res. 2021;20(5):2340–51. 33754726 10.1021/acs.jproteome.0c00852

[22] Caussy C, Hsu C, Singh S, Bassirian S, Kolar J, Faulkner C, et al. Serum bile acid patterns are associated with the presence of NAFLD in twins, and dose-dependent changes with increase in fibrosis stage in patients with biopsy-proven NAFLD. Aliment Pharmacol Ther. 2019;49(2):183–93. 30506692 10.1111/apt.15035 PMC6319963

[23] Kakiyama G, Pandak WM, Gillevet PM, Hylemon PB, Heuman DM, Daita K, et al. Modulation of the fecal bile acid profile by gut microbiota in cirrhosis. J Hepatol. 2013;58(5):949–55. 23333527 10.1016/j.jhep.2013.01.003 PMC3936319

[24] Jones BV, Begley M, Hill C, Gahan CG, Marchesi JR. Functional and comparative metagenomic analysis of bile salt hydrolase activity in the human gut microbiome. Proc Natl Acad Sci USA. 2008;105(36):13580–5. 18757757 10.1073/pnas.0804437105 PMC2533232

[25] Elkins CA, Moser SA, Savage DC. Genes encoding bile salt hydrolases and conjugated bile salt transporters in Lactobacillus johnsonii 100–100 and other Lactobacillus species. Microbiology (Reading). 2001;147(Pt 12):3403–12. 11739773 10.1099/00221287-147-12-3403

[26] Ridlon JM, Devendran S, Alves JM, Doden H, Wolf PG, Pereira GV, et al. The ‘in vivo lifestyle’ of bile acid 7alpha-dehydroxylating bacteria: comparative genomics, metatranscriptomic, and bile acid metabolomics analysis of a defined microbial community in gnotobiotic mice. Gut Microbes. 2020;11(3):381–404. 31177942 10.1080/19490976.2019.1618173 PMC7524365

[27] Jansen PL, Ghallab A, Vartak N, Reif R, Schaap FG, Hampe J, et al. The ascending pathophysiology of cholestatic liver disease. Hepatology. 2017;65(2):722–38. 27981592 10.1002/hep.28965

[28] Chiang JYL, Ferrell JM. Discovery of farnesoid X receptor and its role in bile acid metabolism. Mol Cell Endocrinol. 2022;548: 111618. 35283218 10.1016/j.mce.2022.111618 PMC9038687

[29] Huang L, Zhang Y, Zhang X, Chen X, Wang Y, Lu J, et al. Therapeutic Potential of Pien-Tze-Huang: a review on its chemical composition, pharmacology, and clinical application. Molecules. 2019;24(18):3274. 31505740 10.3390/molecules24183274 PMC6767116

[30] Cao Y, Li T, Chang AQ, Jiang ZZ, Yu J, Tu PF, et al. Bile acid derivatives-focused chemical profiling in snake bile. Zhongguo Zhong Yao Za Zhi. 2021;46(1):130–8. 33645062 10.19540/j.cnki.cjcmm.20200628.204

[31] Chiang JYL. Bile acid metabolism and signaling in liver disease and therapy. Liver Res. 2017;1(1):3–9. 29104811 10.1016/j.livres.2017.05.001 PMC5663306

[32] Falany CN, Johnson MR, Barnes S, Diasio RB. Glycine and taurine conjugation of bile acids by a single enzyme. Molecular cloning and expression of human liver bile acid CoA: amino acid N-acyltransferase. J Biol Chem. 1994;269(30):19375–9. 8034703

[33] Noh K, Kim YM, Kim YW, Kim SG. Farnesoid X receptor activation by chenodeoxycholic acid induces detoxifying enzymes through AMP-activated protein kinase and extracellular signal-regulated kinase 1/2-mediated phosphorylation of CCAAT/enhancer binding protein beta. Drug Metab Dispos. 2011;39(8):1451–9. 21596890 10.1124/dmd.111.038414

[34] Isaacs-Ten A, Echeandia M, Moreno-Gonzalez M, Brion A, Goldson A, Philo M, et al. Intestinal microbiome-macrophage crosstalk contributes to cholestatic liver disease by promoting intestinal permeability in Mice. Hepatology. 2020;72(6):2090–108. 32168395 10.1002/hep.31228 PMC7839474

[35] Zhang P, Li X, Liang J, Zheng Y, Tong Y, Shen J, et al. Chenodeoxycholic acid modulates cholestatic niche through FXR/Myc/P-selectin axis in liver endothelial cells. Nat Commun. 2025;16(1):2093. 40025016 10.1038/s41467-025-57351-2 PMC11873286

[36] Kuang J, Wang J, Li Y, Li M, Zhao M, Ge K, et al. Hyodeoxycholic acid alleviates non-alcoholic fatty liver disease through modulating the gut-liver axis. Cell Metab. 2023;35(10):1752–66. 37591244 10.1016/j.cmet.2023.07.011

[37] Chaudhari SN, Luo JN, Harris DA, Aliakbarian H, Yao L, Paik D, et al. A microbial metabolite remodels the gut-liver axis following bariatric surgery. Cell Host Microbe. 2021;29(3):408–24. 33434516 10.1016/j.chom.2020.12.004 PMC7954942

[38] Pathak P, Xie C, Nichols RG, Ferrell JM, Boehme S, Krausz KW, et al. Intestine farnesoid X receptor agonist and the gut microbiota activate G-protein bile acid receptor-1 signaling to improve metabolism. Hepatology. 2018;68(4):1574–88. 29486523 10.1002/hep.29857 PMC6111007

[39] Zheng X, Chen T, Jiang R, Zhao A, Wu Q, Kuang J, et al. Hyocholic acid species improve glucose homeostasis through a distinct TGR5 and FXR signaling mechanism. Cell Metab. 2021;33(4):791–803. 33338411 10.1016/j.cmet.2020.11.017

