# Supplementary material for: Pien-Tze-Huang alleviates lithocholic acid-induced cholestasis in mice by shaping bile acid-submetabolome
Source: Chin Med. 2025 Jul 3;20:103. doi: 10.1186/s13020-025-01161-7 (PMC12225082; doi:10.1186/s13020-025-01161-7)
Supplement: Supplementary file 1 — Additional file 1 [file 13020_2025_1161_MOESM1_ESM.docx]

**Supporting information**

**Pien-Tze-Huang alleviates lithocholic acid-induced cholestasis in mice by shaping bile acid-submetabolome**

Yan Cao,^1,2†^ Yanhong Zhai,^1†^ Qihong Deng,^3^ Shufen Song,^3^ Wei Li, ^2^ Youran Li,^1^ Yifan Lu, ^1^ Jun Li, ^2^ Zheng Cao^1*^ and Yuelin Song ^2*^

^1^ Department of Laboratory Medicine, Beijing Obstetrics and Gynecology Hospital, Capital Medical University. Beijing Maternal and Child Health Care Hospital, Beijing, China

^2^ Modern Research Center for Traditional Chinese Medicine, Beijing Research Institute of Chinese Medicine, Beijing University of Chinese Medicine, Beijing 100029, China

^3^ Fujian Pien Tze Huang Enterprise Key Laboratory of Natural Medicine Research and Development, Zhangzhou Pien Tze Huang Pharmaceutical Co., Ltd, Zhangzhou 363000, China

^†^ Yan Cao and Yanhong Zhai contributed equally to this work and share first authorship.

* Corresponding Authors

Prof. Zheng Cao, Email: zhengcao2011@ccmu.edu.cn

Prof. Yuelin Song, E-mail: syltwc2005@163.com

**Supplementary Methods**

1. ***In vivo* Protocol**

Male 8-week-old C57BL/6J mice were obtained from the Vital River Laboratory Animal Technology Co., Ltd. (Beijing, China), and the protocols used in these studies were approved by the Animal Ethics Committee of Beijing University of Chinese Medicine (Beijing, China). The animals were housed in a specific-pathogen-free animal facility with a controlled environment (22±°C, 50-60% humidity, 12-hour light-dark cycle, lights on at 7 am) and free access to food and water. After one week of acclimatization, 48 mice were randomly assigned into six groups: control group, model group (LCA-induced cholestasis), UDCA group (150 mg·kg^-1^), low dosage of PTH (PTH-L, 75 mg·kg^-1^), middle dosage of PTH (PTH-M, 150 mg·kg^-1^), and high dosage of PTH (PTH-H, 300 mg·kg^-1^). The mice in the UDCA, PTH-L, PTH-M, and PTH-H groups were administered with corresponding medication continuously for seven days; meanwhile, the control group was administrated with olive oil. From days 4 to 7, the mice in the UDCA, PTH-L, PTH-M, and PTH-H groups received administration of LCA (150 mg·kg^-1^) twice daily for 4 days. On the eighth day, all animals were sacrificed with pentobarbital sodium at the end of the treatment period.

The blood samples (approximately 1 mL) were collected by the abdominal aorta intubation approach in mice. Immediately, the serum was centrifuged at 3000 r·min^-1^ for 10 minutes and separated into two portions. One part of the serum was for used to evaluate hepatic injury indices (ALT, AST, ALP, total bilirubin, and TBA were measured by a fully automatic biochemical analyzer (7180, Hitachi, Japan). Another part of serum was used for widely targeted quantitative bile acids analysis.

For liver perfusion, residual blood was removed with hemostatic forceps, and the liver was immediately removed and divided into two parts. One part of the liver was fixed and embedded in paraffin. Following slicing into 5-μm sections, liver specimens were stained by hematoxylin-eosin and examined in random order. The histological damage in liver tissues was evaluated as described previously^[1]^. One part of the liver was stored at −80°C for subsequent analysis.

The entire intestine was removed and divided into regions (duodenum, jejunum, and ileum). Rubbed with a filter paper to remove the excess water, each region was stored at −80°C for subsequent analysis.

1. **Bile acid analysis**

After thawing at 4°C, 110 μL blood sample was fortified with 330 μL iced ACN containing IS solution. Following 12000 r·min^-1^ centrifugation at 4 °C for 10 min, the supernatants were individually collected and dried under gentle nitrogen blowing. The residues were reconstituted with 50 μL of 50% aqueous ACN prior to LC−MS analysis. Equal volumes of each sample were mixed as quality control (QC) samples. And QC samples were diluted 2 times and 5 times with 50% aqueous ACN, respectively, before subsequent method validation assays.

Right after freezing, approximately 0.3 g of liver tissue or ileum was accurately weighed and then placed in a homogenizer. Three times volume of buffer containing 0.25 M sucrose, 75 mM mannitol, 10 mM Tris-HCl pH 7.4, and 1 mM EDTA was added for homogenization. Subsequently, 150 μL supernatant was collected by centrifugation at 4°C and 12000 r·min^-1^ for 15 min. Three times volume of iced ACN containing IS solution was added and centrifuged at 12000 r·min^-1^ for 10 min to precipitate protein. The supernatants (400 μL) were individually collected and dried under gentle nitrogen blowing. The residues were reconstituted with 50 μL of 50% aqueous ACN prior to LC−MS analysis.

1. **Method validation assays**

Method validation was carried out for a total of 201 compounds (Table S1) that covered all annotated chemical types, in terms of intra- and inter-day, repeatability and stability.

**3.1 Precision**

Calibration samples of low (QC×5), medium (QC×2), and high (QC) dilution levels were selected as quality control samples to assess the intra-day and inter-day precisions. For intra-day variability assessment, all quality control samples were measured for six replicates within a single day, whereas all quality control samples were examined in triplicate per day for three consecutive days to conduct inter-day assay. All the variations were expressed with the relative standard deviations (RSDs%) of the peak area ratios between targeted analytes and internal standard.

**3.2 Repeatability and stability**

To evaluate the repeatability, a selected sample（serum in UDCA group）was run for six replicates using the configured system. Moreover, the sample was deposited at 4°C and analysed every 12 h within three consecutive days to assess the stability.

1. **Untargeted proteomics experiments**

Right after freezing, approximately 0.3 g of liver tissue was accurately weighed and then placed in a homogenizer. Three times the volume of buffer containing 0.25 M sucrose, 75 mM mannitol, 10 mM Tris-HCl pH 7.4, and 1 mM EDTA was added for homogenization. Afterwards, supernatant called S9-fractions was collected for LC−MS analysis by centrifugation at 4°C and 9000 *× g* for 60 min.

1. **Cell culture**

All experiments were conducted in the HepG2 cell line (ATCC). Cultures were maintained in 25 cm^2^ flasks (Corning) in DMEM/F-12 (Gibco #11320033) containing 10% fetal bovine serum (Gibco #A3160402), 1% L-glutamine (Gibco #A2916801), 1% sodium pyruvate (Gibco #11360070), and 1% penicillin-streptomycin (Gibco #15140122) at 5% CO_2_. Cultures were passaged twice per week upon reaching 90% confluency. Approximately 24 h before the intended start of an experiment, cultures were passaged and seeded in a 24-well plate on glass coverslips at a density of approximately 5,000 cells per well.

1. **Illumina sequencing of bacterial 16S rRNA gene**

The raw 16S rRNA gene sequencing reads were demultiplexed, quality filtered by using fastp version 0.20.0, and merged by using FLASH version 1.2.7 with the following criteria^[2,3]^: (i) no contaminant sequences were allowed; (ii) 300-bp reads were truncated at any site receiving an average quality score of <20 over a 50-bp sliding window, with parts of truncated reads shorter than 50 bp and parts of reads containing ambiguous characters both being discarded; (iii) only overlapping sequences longer than 10 bp were assembled, according to their overlapped sequence, with a maximum mismatch ratio of the overlap region of 0.2 and reads that could not be assembled being discarded; and (iv) samples were distinguished according to the barcode and primers, the sequence direction adjusted, exact barcode matching performed, and 2-nucleotide mismatch in primer matching allowed.

**References**

[1] Abo-Haded HM, Elkablawy MA, Al-Johani Z, Al-Ahmadi O, El-Agamy DS. Hepatoprotective effect of sitagliptin against methotrexate induced liver toxicity. PLoS One. 2017 Mar 23;12(3):e0174295.

[2] Chen S, Zhou Y, Chen Y, Gu J. fastp: an ultra-fast all-in-one FASTQ preprocessor. Bioinformatics. 2018 Sep 1;34(17):i884-i890.

[3] Magoč T, Salzberg SL. FLASH: fast length adjustment of short reads to improve genome assemblies. Bioinformatics. 2011 Nov 1;27(21):2957-63.


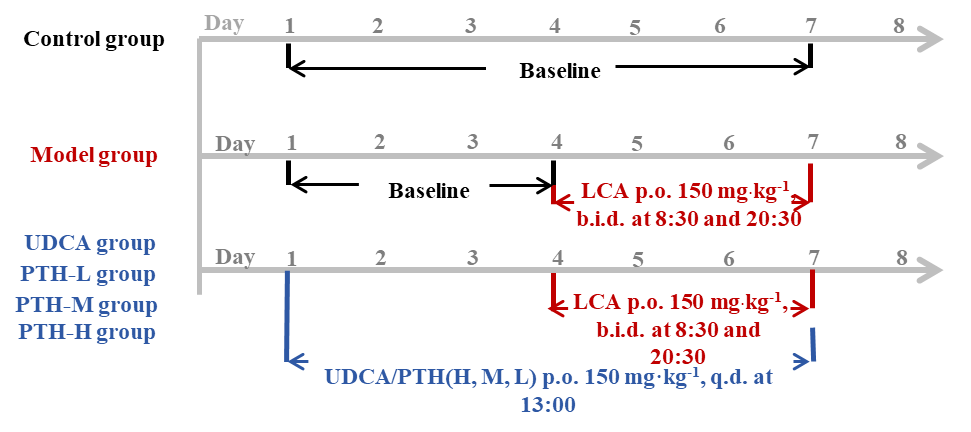


**Figure S1** The detailed flowchart of animal study procedures.


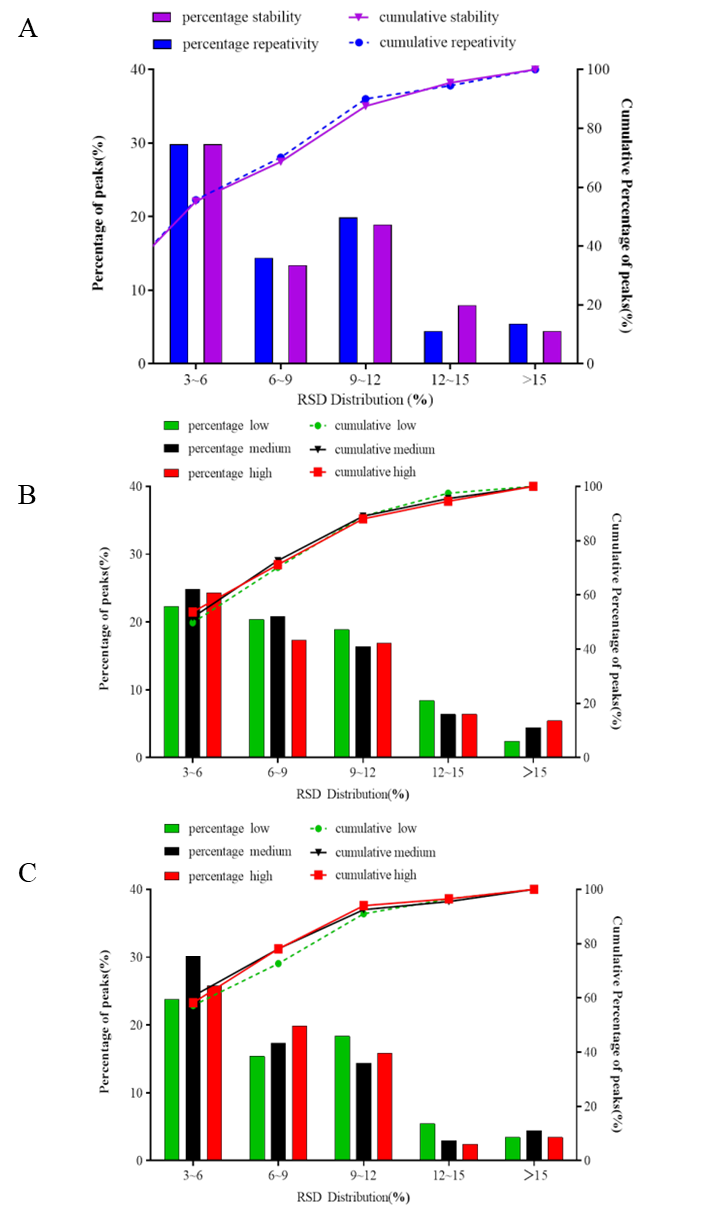


**Fig.S2** Results for diverse method validation assays. A: stability & repeatability; B: intra-day variation; and C: inter-day variation

Table S1 Results for method validation assays.

| Analyte No. | Intra-day  RSD(%, *n* = 6) | | | Inter-day  RSD(%, *n* = 9) | | | Repeatability  RSD  (%, *n* = 6) | Stability  RSD  (%, *n* = 6) |
| --- | --- | --- | --- | --- | --- | --- | --- | --- |
|  | Low | Medium | High | Low | Medium | High |  |  |
| **1** | 3.94 | 2.42 | 1.83 | 3.27 | 8.82 | 2.15 | 4.44 | 0.83 |
| **2** | 1.58 | 8.74 | 4.17 | 6.27 | 2.96 | 8.54 | 5.71 | 12.91 |
| **3** | 7.56 | 3.55 | 3.42 | 11.59 | 10.88 | 9.56 | 6.94 | 3.54 |
| **4** | 7.28 | 4.12 | 2.89 | 8.54 | 9.57 | 12.95 | 3.87 | 1.86 |
| **5** | 3.04 | 6.66 | 8.53 | 2.26 | 9.39 | 1.91 | 0.72 | 3.04 |
| **6** | 1.58 | 8.74 | 10.34 | 0.71 | 8.98 | 1.95 | 13.52 | 13.55 |
| **7** | 7.56 | 3.55 | 10.77 | 10.47 | 9.25 | 4.95 | 22.91 | 8.62 |
| **8** | 7.28 | 4.12 | 10.38 | 19.46 | 4.22 | 10.93 | 3.29 | 9.03 |
| **9** | 10.29 | 10.46 | 5.27 | 2.11 | 0.26 | 5.72 | 0.38 | 11.22 |
| **10** | 3.27 | 11.36 | 12.11 | 6.41 | 7.53 | 1.03 | 0.93 | 10.27 |
| **11** | 6.93 | 5.25 | 5.31 | 9.25 | 8.23 | 3.55 | 13.53 | 4.12 |
| **12** | 6.55 | 13.89 | 0.02 | 1.27 | 2.49 | 3.8 | 5.23 | 9.26 |
| **13** | 9.01 | 10.37 | 2.25 | 0.28 | 2.06 | 0.24 | 8.46 | 1.04 |
| **14** | 4.53 | 7.73 | 2.74 | 7.90 | 5.09 | 6.44 | 6.25 | 4.42 |
| **15** | 8.92 | 5.02 | 11.24 | 10.77 | 3.07 | 6.55 | 2.59 | 7.38 |
| **16** | 5.00 | 3.85 | 5.42 | 10.71 | 8.3 | 4.23 | 3.64 | 10.36 |
| **17** | 4.26 | 1.02 | 14.27 | 19.46 | 0.62 | 1.05 | 2.03 | 9.66 |
| **18** | 9.47 | 10.23 | 2.95 | 0.81 | 5.93 | 10.85 | 1.76 | 8.19 |
| **19** | 0.75 | 9.88 | 21.74 | 2.15 | 6.84 | 20.17 | 10.47 | 10.66 |
| **20** | 2.37 | 12.73 | 0.99 | 7.83 | 6.45 | 8.53 | 5.15 | 10.71 |
| **21** | 0.07 | 0.99 | 0.48 | 4.37 | 0.35 | 9.35 | 1.44 | 9.04 |
| **22** | 2.39 | 7.59 | 2.56 | 1.28 | 1.08 | 1.34 | 2.02 | 4.25 |
| **23** | 8.25 | 10.29 | 10.02 | 3.04 | 5.26 | 0.35 | 9.36 | 6.24 |
| **24** | 4.47 | 1.02 | 3.02 | 5.37 | 3.19 | 0.95 | 7.78 | 1.25 |
| **25** | 5.20 | 6.21 | 8.33 | 9.64 | 2.04 | 7.27 | 3.44 | 0.25 |
| **26** | 2.70 | 8.41 | 4.79 | 2.46 | 5.53 | 7.69 | 4.86 | 12.97 |
| **27** | 10.25 | 4.00 | 5.55 | 2.20 | 6.26 | 3.86 | 6.87 | 4.36 |
| **28** | 14.26 | 7.21 | 3.62 | 8.31 | 9.04 | 6.47 | 10.41 | 11.72 |
| **29** | 10.3 | 2.68 | 0.93 | 1.95 | 3.02 | 3.47 | 3.24 | 3.97 |
| **30** | 14.26 | 3.91 | 4.55 | 5.67 | 3.55 | 7.39 | 12.92 | 13.03 |
| **31** | 0.45 | 9.49 | 0.42 | 2.54 | 1.29 | 10.37 | 11.34 | 9.26 |
| **32** | 0.53 | 0.56 | 6.93 | 8.36 | 11.27 | 10.22 | 7.24 | 9.44 |
| **33** | 14.31 | 5.29 | 7.70 | 2.95 | 0.44 | 1.93 | 5.38 | 4.93 |
| **34** | 7.02 | 4.58 | 8.61 | 0.38 | 7.02 | 1.22 | 9.94 | 4.03 |
| **35** | 5.24 | 10.55 | 9.59 | 12.73 | 0.94 | 3.29 | 4.83 | 0.45 |
| **36** | 6.44 | 5.64 | 8.24 | 2.84 | 4.29 | 4.53 | 3.04 | 4.39 |
| **37** | 9.82 | 4.87 | 2.41 | 1.11 | 2.40 | 0.48 | 3.22 | 7.01 |
| **38** | 2.01 | 11.77 | 8.93 | 2.32 | 4.91 | 7.82 | 3.92 | 5.93 |
| **39** | 4.28 | 13.43 | 14.38 | 1.04 | 11.27 | 10.31 | 4.05 | 5.39 |

Table S1 Results for method validation assays. (continued)

| Analyte No. | Intra-day  RSD(%, *n* = 6) | | | Inter-day  RSD(%, *n* = 9) | | | Repeatability  RSD  (%, *n* = 6) | Stability  RSD  (%, *n* = 6) |
| --- | --- | --- | --- | --- | --- | --- | --- | --- |
|  | Low | Medium | High | Low | Medium | High |  |  |
| **40** | 8.42 | 7.32 | 8.56 | 4.34 | 6.35 | 5.33 | 9.05 | 7.28 |
| **41** | 6.64 | 11.31 | 12.76 | 10.34 | 0.83 | 1.47 | 7.36 | 2.83 |
| **42** | 9.90 | 1.05 | 9.27 | 8.65 | 6.34 | 5.98 | 3.83 | 3.22 |
| **43** | 10.38 | 2.18 | 5.29 | 7.92 | 10.42 | 9.47 | 6.96 | 12.75 |
| **44** | 12.88 | 3.05 | 9.55 | 9.03 | 11.35 | 4.59 | 4.24 | 0.28 |
| **45** | 8.31 | 9.09 | 9.37 | 7.25 | 5.82 | 2.33 | 10.52 | 3.29 |
| **46** | 3.53 | 5.15 | 4.27 | 4.79 | 1.38 | 6.82 | 5.36 | 11.59 |
| **47** | 4.44 | 14.22 | 10.65 | 5.55 | 0.01 | 8.35 | 9.63 | 0.28 |
| **48** | 3.29 | 8.55 | 0.82 | 2.82 | 3.41 | 2.04 | 5.25 | 2.93 |
| **49** | 8.56 | 9.98 | 8.57 | 4.50 | 9.46 | 0.95 | 3.77 | 2.02 |
| **50** | 0.52 | 12.53 | 10.23 | 4.28 | 5.62 | 1.21 | 2.31 | 4.75 |
| **51** | 5.35 | 8.92 | 2.74 | 7.21 | 2.84 | 4.28 | 0.91 | 10.38 |
| **52** | 0.93 | 12.47 | 8.55 | 2.01 | 3.39 | 8.96 | 9.32 | 13.92 |
| **53** | 11.46 | 0.04 | 4.93 | 4.38 | 2.91 | 5.29 | 13.42 | 8.43 |
| **54** | 0.92 | 8.04 | 5.38 | 9.66 | 3.43 | 1.31 | 11.29 | 7.38 |
| **55** | 10.42 | 6.25 | 3.74 | 0.81 | 3.61 | 11.27 | 1.79 | 10.34 |
| **56** | 11.38 | 7.06 | 10.85 | 5.42 | 2.50 | 0.85 | 2.75 | 11.28 |
| **57** | 10.94 | 8.83 | 0.92 | 5.13 | 12.54 | 10.26 | 6.35 | 0.03 |
| **58** | 2.48 | 0.96 | 8.17 | 5.02 | 2.47 | 3.04 | 8.54 | 6.40 |
| **59** | 5.32 | 2.59 | 4.93 | 9.27 | 0.72 | 0.16 | 9.56 | 1.14 |
| **60** | 7.41 | 5.62 | 0.71 | 3.52 | 1.63 | 4.72 | 10.52 | 1.11 |
| **61** | 2.49 | 4.24 | 0.85 | 13.17 | 4.95 | 11.38 | 2.55 | 2.44 |
| **62** | 4.21 | 10.82 | 1.38 | 8.75 | 4.28 | 9.47 | 4.28 | 5.37 |
| **63** | 9.33 | 2.01 | 3.91 | 2.46 | 10.42 | 3.21 | 0.28 | 8.31 |
| **64** | 0.84 | 4.36 | 2.10 | 5.62 | 6.66 | 4.66 | 0.08 | 2.47 |
| **65** | 10.42 | 2.03 | 4.73 | 1.21 | 3.52 | 9.39 | 3.72 | 1.92 |
| **66** | 11.26 | 5.38 | 2.44 | 1.36 | 4.95 | 0.46 | 6.83 | 3.26 |
| **67** | 14.25 | 8.91 | 2.19 | 0.81 | 6.10 | 10.24 | 2.21 | 10.15 |
| **68** | 5.63 | 9.99 | 4.1 | 1.30 | 5.94 | 2.86 | 0.68 | 1.36 |
| **69** | 10.93 | 2.24 | 7.16 | 1.93 | 3.90 | 11.08 | 6.32 | 9.33 |
| **70** | 1.21 | 13.52 | 11.27 | 5.35 | 8.62 | 2.63 | 9.27 | 3.26 |
| **71** | 7.43 | 4.11 | 10.65 | 2.62 | 10.36 | 2.18 | 8.29 | 4.28 |
| **72** | 1.30 | 0.48 | 9.47 | 2.48 | 0.87 | 2.10 | 0.81 | 3.25 |
| **73** | 5.05 | 6.99 | 4.59 | 2.03 | 3.82 | 12.97 | 0.38 | 0.94 |
| **74** | 4.86 | 4.93 | 8.63 | 2.18 | 4.42 | 0.38 | 4.58 | 1.86 |
| **75** | 5.09 | 2.59 | 3.86 | 3.69 | 0.48 | 6.82 | 5.55 | 4.24 |
| **76** | 5.70 | 3.92 | 3.96 | 5.48 | 5.37 | 1.82 | 0.58 | 7.83 |
| **77** | 9.30 | 7.39 | 1.22 | 13.81 | 1.20 | 0.99 | 6.71 | 5.52 |
| **78** | 4.93 | 2.84 | 9.95 | 10.47 | 14.91 | 4.84 | 4.28 | 1.03 |

Table S1 Results for method validation assays. (continued)

| Analyte No. | Intra-day  RSD(%, *n* = 6) | | | Inter-day  RSD(%, *n* = 9) | | | Repeatability  RSD  (%, *n* = 6) | Stability  RSD  (%, *n* = 6) |
| --- | --- | --- | --- | --- | --- | --- | --- | --- |
|  | Low | Medium | High | Low | Medium | High |  |  |
| **79** | 4.22 | 8.27 | 11.79 | 7.97 | 3.92 | 2.77 | 3.69 | 3.61 |
| **80** | 4.92 | 4.22 | 2.19 | 9.82 | 5.40 | 2.05 | 8.57 | 7.82 |
| **77** | 9.30 | 7.39 | 1.22 | 13.81 | 1.20 | 0.99 | 6.71 | 5.52 |
| **78** | 4.93 | 2.84 | 9.95 | 10.47 | 14.91 | 4.84 | 4.28 | 1.03 |
| **79** | 4.22 | 8.27 | 11.79 | 7.97 | 3.92 | 2.77 | 3.69 | 3.61 |
| **80** | 4.92 | 4.22 | 2.19 | 9.82 | 5.40 | 2.05 | 8.57 | 7.82 |
| **81** | 1.02 | 5.17 | 5.11 | 9.61 | 6.73 | 10.31 | 3.95 | 5.76 |
| **82** | 2.77 | 19.75 | 6.33 | 3.50 | 6.84 | 0.84 | 11.27 | 15.69 |
| **83** | 1.20 | 4.90 | 12.3 | 1.73 | 2.44 | 2.61 | 0.94 | 7.35 |
| **84** | 14.92 | 14.89 | 6.77 | 5.20 | 9.75 | 6.58 | 10.83 | 13.2 |
| **85** | 4.77 | 11.77 | 6.58 | 22.94 | 3.25 | 3.70 | 2.04 | 8.04 |
| **86** | 0.84 | 11.91 | 0.87 | 2.11 | 4.93 | 6.91 | 7.53 | 7.72 |
| **87** | 11.62 | 9.97 | 17.06 | 9.21 | 2.20 | 9.92 | 13.98 | 14.19 |
| **88** | 1.28 | 3.80 | 0.06 | 10.38 | 1.29 | 3.10 | 10.83 | 1.86 |
| **89** | 10.31 | 3.32 | 8.77 | 7.35 | 6.82 | 0.73 | 8.30 | 4.44 |
| **90** | 2.81 | 2.49 | 0.84 | 1.03 | 4.82 | 4.92 | 3.60 | 9.62 |
| **91** | 3.20 | 0.84 | 2.61 | 10.33 | 3.88 | 4.29 | 3.73 | 9.41 |
| **92** | 3.70 | 3.82 | 6.58 | 0.95 | 5.78 | 6.45 | 7.13 | 10.27 |
| **93** | 7.53 | 3.59 | 2.66 | 1.66 | 5.99 | 5.97 | 8.77 | 4.99 |
| **94** | 13.98 | 5.39 | 7.87 | 2.49 | 2.46 | 1.26 | 11.61 | 1.04 |
| **95** | 10.83 | 1.27 | 6.75 | 4.88 | 0.92 | 8.30 | 12.26 | 7.19 |
| **96** | 8.30 | 2.94 | 12.32 | 2.91 | 12.61 | 0.83 | 0.83 | 2.47 |
| **97** | 7.39 | 0.72 | 1.40 | 5.71 | 3.30 | 11.83 | 5.72 | 1.83 |
| **98** | 2.84 | 6.33 | 0.38 | 7.72 | 4.94 | 0.99 | 10.11 | 13.41 |
| **99** | 6.11 | 5.94 | 12.16 | 10.28 | 6.25 | 6.82 | 14.19 | 0.81 |
| **100** | 8.55 | 5.64 | 1.83 | 0.92 | 2.61 | 6.26 | 4.98 | 3.28 |
| **101** | 8.91 | 7.84 | 0.93 | 11.31 | 4.02 | 8.98 | 8.47 | 0.73 |
| **102** | 8.04 | 5.60 | 8.56 | 3.51 | 6.29 | 2.29 | 10.94 | 3.17 |
| **103** | 1.03 | 4.27 | 0.83 | 10.27 | 0.07 | 3.26 | 0.06 | 7.32 |
| **104** | 5.28 | 11.12 | 9.37 | 0.20 | 2.20 | 7.32 | 5.00 | 7.35 |
| **105** | 0.07 | 1.20 | 6.73 | 9.17 | 0.50 | 6.03 | 9.48 | 4.20 |
| **106** | 12.16 | 0.84 | 2.30 | 9.20 | 0.81 | 5.94 | 10.16 | 0.73 |
| **107** | 0.96 | 11.11 | 5.88 | 4.29 | 10.28 | 1.65 | 12.26 | 7.39 |
| **108** | 0.37 | 0.82 | 2.47 | 13.61 | 9.77 | 21.46 | 5.33 | 10.22 |
| **109** | 13.61 | 6.33 | 10.28 | 0.48 | 6.76 | 2.10 | 18.63 | 14.92 |
| **110** | 7.41 | 7.87 | 2.17 | 3.33 | 8.86 | 5.83 | 2.40 | 0.26 |
| **111** | 5.59 | 6.75 | 9.66 | 14.82 | 15.35 | 3.77 | 9.01 | 14.82 |
| **112** | 7.18 | 12.32 | 7.45 | 3.31 | 5.37 | 0.28 | 10.83 | 5.28 |
| **113** | 6.32 | 7.45 | 7.33 | 4.25 | 12.65 | 2.01 | 3.48 | 5.28 |

Table S1 Results for method validation assays. (continued)

| Analyte No. | Intra-day  RSD(%, *n* = 6) | | | Inter-day  RSD(%, *n* = 9) | | | Repeatability  RSD  (%, *n* = 6) | Stability  RSD  (%, *n* = 6) |
| --- | --- | --- | --- | --- | --- | --- | --- | --- |
|  | Low | Medium | High | Low | Medium | High |  |  |
| **114** | 8.20 | 13.67 | 13.52 | 2.21 | 4.39 | 11.38 | 9.72 | 5.55 |
| **115** | 13.79 | 7.19 | 19.46 | 1.05 | 1.37 | 2.28 | 2.18 | 10.84 |
| **116** | 8.82 | 11.56 | 11.48 | 8.88 | 0.76 | 0.04 | 10.42 | 11.42 |
| **117** | 14.95 | 11.31 | 17.26 | 4.29 | 10.84 | 10.27 | 10.33 | 0.03 |
| **118** | 13.08 | 8.46 | 7.84 | 0.95 | 11.83 | 7.51 | 5.09 | 2.18 |
| **119** | 11.35 | 13.66 | 10.92 | 6.29 | 7.53 | 10.22 | 10.99 | 13.98 |
| **120** | 4.85 | 4.47 | 5.29 | 0.82 | 17.46 | 8.36 | 1.20 | 4.71 |
| **121** | 6.38 | 10.27 | 4.87 | 11.26 | 23.84 | 0.99 | 9.75 | 1.29 |
| **122** | 10.14 | 0.86 | 12.84 | 12.98 | 10.82 | 0.72 | 7.59 | 4.23 |
| **123** | 5.72 | 2.18 | 4.86 | 10.5 | 0.58 | 5.22 | 0.95 | 7.73 |
| **124** | 3.28 | 1.29 | 5.31 | 9.68 | 2.95 | 16.27 | 10.75 | 9.74 |
| **125** | 4.29 | 0.40 | 0.27 | 2.18 | 11.11 | 10.84 | 2.85 | 6.41 |
| **126** | 11.27 | 7.97 | 10.95 | 6.39 | 7.72 | 1.27 | 1.04 | 18.11 |
| **127** | 0.81 | 4.54 | 2.85 | 4.27 | 6.20 | 7.83 | 10.06 | 9.61 |
| **128** | 3.18 | 2.42 | 9.05 | 8.56 | 12.94 | 4.65 | 6.32 | 0.33 |
| **129** | 2.10 | 4.52 | 0.55 | 2.75 | 7.55 | 3.05 | 8.79 | 3.17 |
| **130** | 0.83 | 5.53 | 3.32 | 12.04 | 1.39 | 9.58 | 1.05 | 0.72 |
| **131** | 8.87 | 2.06 | 2.36 | 0.48 | 8.95 | 9.22 | 5.81 | 4.91 |
| **132** | 7.04 | 1.75 | 23.61 | 6.93 | 15.82 | 3.77 | 1.05 | 4.88 |
| **133** | 10.21 | 7.58 | 3.37 | 0.62 | 9.30 | 2.95 | 1.17 | 3.18 |
| **134** | 2.05 | 4.48 | 3.31 | 0.89 | 7.77 | 8.71 | 5.04 | 5.22 |
| **135** | 1.93 | 8.07 | 3.34 | 4.48 | 3.92 | 9.68 | 2.54 | 4.10 |
| **136** | 7.15 | 5.33 | 3.50 | 8.07 | 3.09 | 6.29 | 2.42 | 0.84 |
| **137** | 4.98 | 9.55 | 7.59 | 5.33 | 7.22 | 3.15 | 5.91 | 8.15 |
| **138** | 3.04 | 2.95 | 3.22 | 0.91 | 2.45 | 4.92 | 10.88 | 10.55 |
| **139** | 4.85 | 7.59 | 2.18 | 9.73 | 5.33 | 5.29 | 4.87 | 25.72 |
| **140** | 7.57 | 3.58 | 21.40 | 2.05 | 3.56 | 3.63 | 18.82 | 3.92 |
| **141** | 13.71 | 2.03 | 21.29 | 11.48 | 4.99 | 4.01 | 15.8 | 2.04 |
| **142** | 3.62 | 5.35 | 13.83 | 2.04 | 4.20 | 4.82 | 1.99 | 0.83 |
| **143** | 1.59 | 9.49 | 10.92 | 13.11 | 6.02 | 0.84 | 15.27 | 10.99 |
| **144** | 10.27 | 2.42 | 10.27 | 3.10 | 23.59 | 0.58 | 8.26 | 5.38 |
| **145** | 6.73 | 2.08 | 2.28 | 10.91 | 2.17 | 0.05 | 2.88 | 5.73 |
| **146** | 2.16 | 18.61 | 8.65 | 3.66 | 10.50 | 4.14 | 7.40 | 13.82 |
| **147** | 18.26 | 2.07 | 10.83 | 15.92 | 13.29 | 2.02 | 3.20 | 0.96 |
| **148** | 2.99 | 19.36 | 3.85 | 3.30 | 5.05 | 4.03 | 4.77 | 1.74 |
| **149** | 19.27 | 0.61 | 1.37 | 2.94 | 4.44 | 3.64 | 5.78 | 1.07 |
| **150** | 13.84 | 19.15 | 0.95 | 10.82 | 6.28 | 10.26 | 3.82 | 2.32 |
| **151** | 0.90 | 10.04 | 0.28 | 13.74 | 3.86 | 1.36 | 10.79 | 10.36 |
| **152** | 2.47 | 2.97 | 12.84 | 3.02 | 5.77 | 14.21 | 7.63 | 20.71 |

Table S1 Results for method validation assays. (continued)

| Analyte No. | Intra-day  RSD(%, *n* = 6) | | | Inter-day  RSD(%, *n* = 9) | | | Repeatability  RSD  (%, *n* = 6) | Stability  RSD  (%, *n* = 6) |
| --- | --- | --- | --- | --- | --- | --- | --- | --- |
|  | Low | Medium | High | Low | Medium | High |  |  |
| **153** | 0.29 | 8.85 | 3.35 | 8.53 | 9.95 | 2.88 | 12.52 | 14.28 |
| **154** | 2.92 | 6.19 | 0.82 | 9.88 | 4.05 | 8.95 | 1.37 | 12.15 |
| **155** | 5.87 | 8.00 | 3.18 | 6.58 | 4.33 | 6.99 | 5.76 | 4.82 |
| **156** | 2.50 | 8.52 | 3.02 | 2.84 | 4.66 | 24.71 | 3.06 | 1.86 |
| **157** | 0.93 | 7.96 | 1.28 | 0.71 | 1.63 | 8.95 | 4.39 | 3.22 |
| **158** | 2.39 | 5.00 | 2.63 | 9.72 | 10.62 | 15.63 | 2.84 | 4.23 |
| **159** | 0.50 | 1.38 | 2.22 | 20.74 | 9.01 | 6.92 | 27.72 | 4.41 |
| **160** | 8.52 | 5.20 | 6.25 | 6.39 | 3.77 | 0.63 | 15.94 | 3.06 |
| **161** | 2.06 | 4.88 | 5.81 | 3.37 | 0.95 | 3.40 | 7.28 | 6.02 |
| **162** | 10.64 | 20.22 | 16.18 | 17.00 | 22.68 | 4.29 | 3.75 | 4.03 |
| **163** | 26.38 | 19.27 | 2.92 | 4.59 | 7.89 | 6.66 | 9.02 | 19.25 |
| **164** | 4.76 | 0.73 | 17.29 | 2.93 | 3.31 | 10.04 | 10.2 | 6.87 |
| **165** | 4.89 | 10.28 | 3.44 | 5.39 | 2.04 | 4.72 | 0.94 | 3.74 |
| **166** | 3.37 | 2.40 | 9.61 | 13.52 | 0.97 | 0.93 | 0.62 | 3.06 |
| **167** | 14.55 | 12.13 | 4.23 | 3.81 | 3.74 | 3.95 | 5.72 | 16.04 |
| **168** | 9.11 | 4.46 | 3.81 | 10.28 | 10.28 | 7.06 | 2.29 | 2.86 |
| **169** | 1.47 | 8.72 | 10.11 | 1.06 | 1.05 | 14.28 | 20.11 | 3.37 |
| **170** | 8.90 | 1.39 | 1.55 | 8.67 | 0.73 | 3.50 | 9.88 | 3.02 |
| **171** | 10.04 | 0.22 | 6.60 | 4.21 | 1.95 | 2.10 | 4.06 | 4.27 |
| **172** | 24.01 | 1.05 | 4.22 | 0.90 | 2.05 | 10.16 | 1.29 | 2.59 |
| **173** | 9.57 | 2.21 | 8.81 | 2.40 | 2.63 | 3.84 | 5.75 | 10.28 |
| **174** | 11.34 | 2.21 | 3.21 | 8.73 | 20.61 | 2.52 | 2.48 | 0.42 |
| **175** | 1.12 | 0.26 | 2.44 | 8.20 | 3.72 | 2.25 | 6.57 | 10.85 |
| **176** | 9.74 | 0.51 | 11.61 | 14.29 | 10.37 | 2.91 | 5.00 | 0.54 |
| **177** | 8.09 | 10.16 | 6.92 | 10.11 | 10.88 | 7.20 | 3.70 | 4.70 |
| **178** | 1.11 | 17.04 | 13.72 | 2.10 | 2.80 | 3.99 | 5.93 | 6.86 |
| **179** | 1.66 | 3.99 | 10.27 | 1.21 | 2.84 | 8.74 | 5.17 | 19.43 |
| **180** | 13.31 | 8.92 | 0.38 | 2.19 | 21.85 | 0.72 | 1.69 | 10.83 |
| **181** | 9.22 | 1.29 | 8.26 | 1.28 | 1.04 | 4.60 | 5.76 | 3.17 |
| **182** | 19.44 | 10.27 | 8.74 | 11.49 | 3.26 | 7.02 | 5.71 | 3.18 |
| **183** | 14.05 | 4.97 | 2.31 | 9.35 | 1.20 | 6.04 | 1.20 | 0.29 |
| **184** | 1.07 | 3.49 | 2.47 | 10.20 | 11.34 | 10.38 | 4.25 | 22.81 |
| **185** | 9.15 | 8.29 | 12.63 | 4.91 | 4.70 | 9.55 | 3.70 | 8.30 |
| **186** | 11.28 | 7.53 | 4.30 | 9.57 | 1.33 | 2.00 | 2.69 | 6.74 |
| **187** | 4.21 | 16.29 | 4.99 | 11.26 | 2.07 | 6.46 | 5.15 | 4.70 |
| **188** | 9.11 | 10.48 | 20.41 | 0.86 | 5.83 | 6.28 | 15.97 | 11.15 |
| **189** | 10.88 | 17.82 | 9.22 | 5.88 | 3.44 | 10.35 | 0.71 | 2.19 |
| **190** | 2.08 | 2.92 | 9.20 | 4.62 | 3.59 | 6.47 | 20.04 | 9.73 |
| **191** | 4.34 | 2.28 | 18.02 | 5.27 | 8.41 | 2.18 | 19.05 | 10.23 |

Table S1 Results for method validation assays. (continued)

| Analyte No. | Intra-day  RSD(%, *n* = 6) | | | Inter-day  RSD(%, *n* = 9) | | | Repeatability  RSD  (%, *n* = 6) | Stability  RSD  (%, *n* = 6) |
| --- | --- | --- | --- | --- | --- | --- | --- | --- |
|  | Low | Medium | High | Low | Medium | High |  |  |
| **192** | 2.04 | 0.42 | 1.48 | 0.91 | 5.66 | 2.33 | 9.20 | 3.86 |
| **193** | 9.57 | 10.58 | 0.98 | 8.63 | 8.11 | 14.72 | 1.26 | 19.47 |
| **194** | 2.04 | 9.33 | 3.27 | 6.83 | 22.25 | 3.28 | 8.25 | 0.71 |
| **195** | 8.29 | 2.39 | 0.71 | 8.40 | 2.79 | 0.74 | 0.94 | 4.41 |
| **196** | 1.32 | 9.24 | 1.23 | 2.01 | 8.33 | 3.03 | 10.26 | 13.89 |
| **197** | 6.33 | 6.65 | 7.92 | 20.38 | 8.63 | 4.44 | 11.84 | 1.25 |
| **198** | 8.77 | 10.52 | 3.39 | 4.93 | 2.70 | 10.82 | 9.57 | 11.91 |
| **199** | 11.74 | 13.23 | 9.01 | 4.44 | 9.20 | 0.95 | 0.44 | 1.22 |
| **200** | 4.05 | 5.33 | 4.53 | 8.41 | 3.81 | 19.02 | 10.22 | 9.36 |
| **201** | 8.36 | 8.24 | 8.52 | 3.72 | 1.11 | 20.61 | 0.83 | 10.37 |


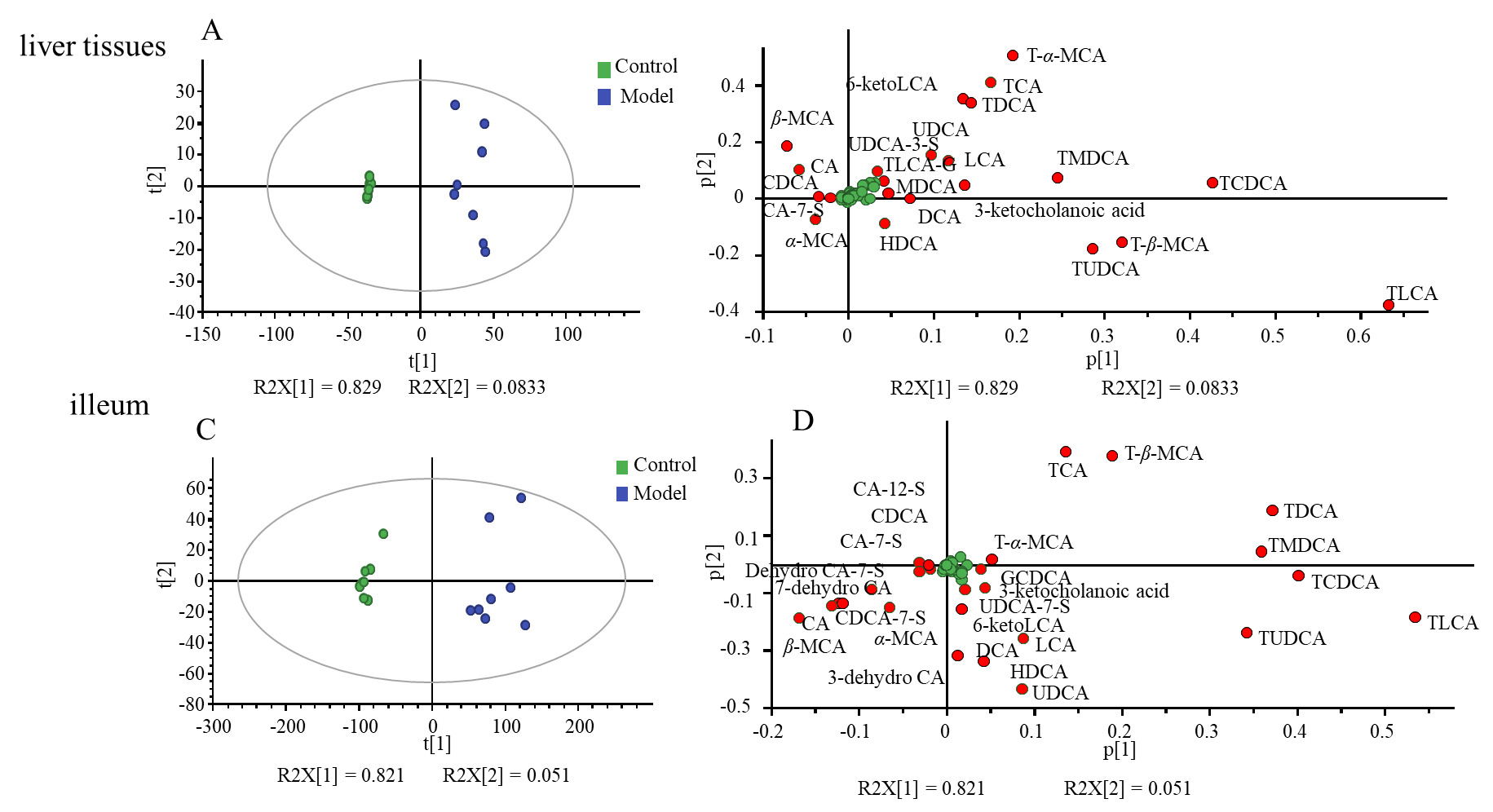


**Fig. S3** Score scattering (A and C) and loading (B and D) plots generated from principal component analysis of samples from both control and model group.


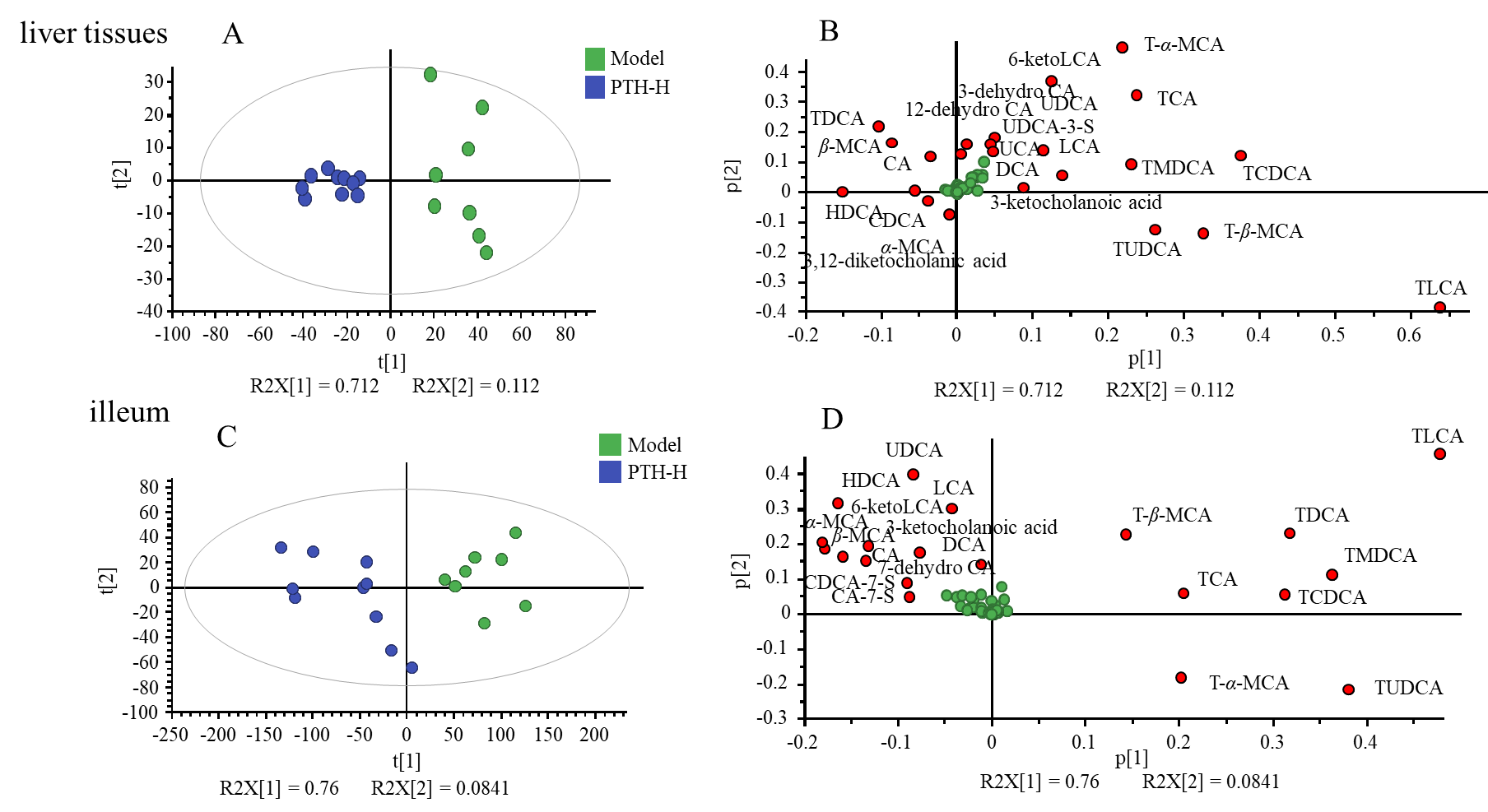


**Fig. S4** Score scattering (A and C) and loading (B and D) plots generated from principal component analysis of samples from both model and PTH-H group.

**Table S2** 38 proteins tentatively annotated as CYP450s, SULTs, and UGTs.

| Protein IDs | Peptide counts (all) | Peptide counts (unique) | Protein names | Gene names | Best MS/MS | control vs. model | UDCA vs. model | PTH vs. model |
| --- | --- | --- | --- | --- | --- | --- | --- | --- |
| A2A977;O88833;A2A8T1;F8WGU9;A0A087WPC3 | 7;7;4;3;1 | 6;6;4;2;1 | Cytochrome P450 4A10 | Cyp4a10;Cyp4a32 | 31453;47302;54539;56041;127077;134794;201132 | ↓ | ↓ | ↑ |
| E9Q5K4 | 8 | 7 |  | Cyp2c44 | 36725;40071;42105;55446;72736;74263;188068;208064 | ↓ | ↑ | ↑ |
| O09158;D3Z2W7;D3YYZ0;D3Z707;D6RJM4 | 5;3;2;1;1 | 5;3;2;1;1 | Cytochrome P450 3A25 | Cyp3a25;Cyp3a59 | 8562;33386;48995;119533;150280 | ↓ | ↑ | ↑ |
| O88962 | 5 | 5 | 7-alpha-hydroxycholest-4-en-3-one 12-alpha-hydroxylase | Cyp8b1 | 55207;148065;151633;200630;202014 | ↓*** | ↑*** | ↑*** |
| P00186;P00184 | 15;4 | 15;4 | Cytochrome P450 1A2 | Cyp1a2 | 23759;33078;37008;59742;86775;87004;100552;101886;104280;122152;137560;138694;159936;179234;209029 | ↓*** | ↑** | ↑** |
| P11714 | 15 | 7 | Cytochrome P450 2D9 | Cyp2d9 | 22279;56235;56744;62549;72146;74548;97319;101894;136630;151056;151882;155038;158073;177853;196388 | ↓ | ↑ | ↓ |
| Q91X75;P20852;P15392;F7B9W9 | 8;8;5;1 | 7;7;5;1 | Cytochrome P450 2A5;Cytochrome P450 2A4 | Cyp2a5;Cyp2a4 | 22705;36921;61892;66527;79459;94408;163630;172590 | ↓* | ↑ | ↑* |
| P24456 | 16 | 5 | Cytochrome P450 2D10 | Cyp2d10 | 22352;25762;56245;62549;71936;73865;79506;101894;136630;139398;151083;151878;158073;158734;190399;196328 | ↓ | ↑ | ↑ |
| P56593;B2RXZ2 | 15;7 | 14;7 | Cytochrome P450 2A12 | Cyp2a12 | 22684;23084;23111;36845;61892;66534;74434;79472;106461;131068;138488;147548;166765;209011;212335 | ↓*** | ↑ | ↑* |
| P56654 | 10 | 2 | Cytochrome P450 2C37 | Cyp2c37 | 23022;31670;36875;55404;57653;74909;90057;119007;131059;195981 | ↑ | ↓ | ↓*** |
| Q05421 | 12 | 12 | Cytochrome P450 2E1 | Cyp2e1 | 25623;36366;36432;52556;55668;58000;61926;65471;98229;112106;130777;209738 | ↓ | ↑ | ↑ |
| Q60991 | 2 | 2 | 25-hydroxycholesterol 7-alpha-hydroxylase | Cyp7b1 | 45355;96570 | ↓ | ↑ | ↓** |
| Q64458;Q3UT49;H3BLM0 | 9;8;4 | 8;7;4 | Cytochrome P450 2C29 | Cyp2c29 | 27237;36672;39961;50560;65996;73691;74895;119006;156758 | ↓*** | ↑** | ↑*** |
| Q64459;Q9JMA7;Q64481 | 13;6;1 | 8;2;0 | Cytochrome P450 3A11 | Cyp3a11 | 8474;10887;22656;33413;47729;99292;101100;114389;119502;127023;144550;179412;183103 | ↓* | ↑ | ↑ |
| Q64464 | 14 | 13 | Cytochrome P450 3A13 | Cyp3a13 | 8463;8473;10908;47720;51168;54400;57164;119490;127019;144460;144576;147946;167788;212571 | ↑ | ↑ | ↑ |
| Q64505 | 1 | 1 | Cholesterol 7-alpha-monooxygenase | Cyp7a1 | 156913 | ↓* | ↑ | ↑* |
| Q6XVG2;Q148B1 | 8;1 | 4;0 | Cytochrome P450 2C54 | Cyp2c54 | 23012;31649;40071;50543;55404;64840;119007;195981 | ↓ | ↑ | ↑ |
| Q8CIM7;Q6P8N9 | 15;2 | 10;0 | Cytochrome P450 2D26 | Cyp2d26 | 22352;29207;35280;54032;56203;61075;61760;62549;64099;72217;74806;75307;79506;150998;188456 | ↑ | ↑ | ↑ |
| Q8K0C4 | 5 | 5 | Lanosterol 14-alpha demethylase | Cyp51a1 | 48832;105427;124083;133119;160873 | ↑* | ↓ | ↓* |
| Q91W64 | 10 | 9 | Cytochrome P450 2C70 | Cyp2c70 | 36636;40071;55371;55669;57865;94421;102089;141883;177587;207839 | ↑ | ↓** | ↓ |
| Q91X77;Q91X77-2 | 11;10 | 3;3 | Cytochrome P450 2C50 | Cyp2c50 | 23022;31670;36875;40071;55404;61961;74625;90057;119011;156758;195981 | ↓ | ↑ | ↑*** |
| Q9DBG1 | 10 | 10 | Sterol 26-hydroxylase, mitochondrial | Cyp27a1 | 15048;22426;31008;51165;52919;97808;127088;141629;160625;204842 | ↓*** | ↓ | ↑* |
| Q9QWG7;Q9QWG7-2 | 2;2 | 2;2 | Sulfotransferase family cytosolic 1B member 1 | Sult1b1 | 83887;170711 | ↓ | ↑ | ↑** |
| E9QNL5;P52840;D3Z3G5;D3Z2P8 | 6;6;3;1 | 1;1;0;0 | Sulfotransferase;Sulfotransferase 1A1 | Sult1a1 | 74546;90842;100413;139077;159165;170738 | ↑*** | ↑ | ↑** |
| Q3UZZ6 | 3 | 3 | Sulfotransferase 1 family member D1 | Sult1d1 | 26410;87269;137842 | ↓* | ↑* | ↑** |
| Q8BGL3;E9QAK1;Q3UEP5;D3Z6M7;L7N245;K7N6K9;P50236 | 11;7;1;1;1;1;1 | 10;7;0;0;0;0;0 | Sulfotransferase | 2810007J24Rik | 451;22003;48349;63817;65643;98522;100282;117152;134440;135247;175380 | ↓* | ↑ |  |
| B2RT14;P70691 | 5;5 | 1;1 | UDP-glucuronosyltransferase 1-2 | Ugt1a5;Ugt1a2 | 12356;64653;93913;121252;202618 |  |  |  |
| H3BLE9;Q3UP75 | 1;1 | 1;1 | UDP-glucuronosyltransferase 3A1 | Ugt3a1 | 117085 |  |  |  |
| K9J7B2;D3YZE6;D3Z7G0;D3YZ96;G3UZC9;D6RH94 | 9;4;4;4;3;1 | 2;0;0;0;0;0 |  | Ugt1a6b | 12356;43621;61626;64653;93913;137863;202618;204137;204848 | ↓*** | ↑ | ↑*** |
| Q8K169;P17717;Q91WH2;Q8VCN3 | 9;9;8;5 | 6;6;5;2 | UDP-glucuronosyltransferase 2B17 | Ugt2b5;Ugt2b17;Ugt2b38;Ugt2b37 | 30355;56167;56191;62072;68484;88466;153654;174699;202618 | ↓* | ↑*** | ↑* |
| Q3UEP4;D3YUP6;D3YWN0;D3YXD5 | 8;6;4;3 | 5;3;2;2 |  | Ugt2b36 | 68484;88378;141819;141861;153382;174748;202618;203212 | ↓ | ↑* | ↑ |
| Q62452;E9PXN7;D3Z748;Q6ZQM8 | 9;6;5;5 | 5;2;1;1 | UDP-glucuronosyltransferase 1-9;UDP-glucuronosyltransferase 1-7C | Ugt1a9;Ugt1a10;Ugt1a8;Ugt1a7c | 12356;64653;93913;155204;167400;174905;180700;202618;211343 | ↑ | ↑ | ↓ |
| Q63886 | 7 | 3 | UDP-glucuronosyltransferase 1-1 | Ugt1a1 | 12356;64653;68497;93913;159459;166152;202618 | ↑ | ↑ | ↑* |
| Q8BJL9 | 9 | 5 |  | Ugt2b35 | 34283;62086;68484;88466;124356;141861;153384;174652;202618 | ↑ | ↑ | ↑ |
| Q8BWQ1 | 9 | 7 | UDP-glucuronosyltransferase 2A3 | Ugt2a3 | 2780;18950;68477;87134;93795;109270;171700;196999;209964 | ↑ | ↑* | ↑ |
| Q8JZZ0 | 5 | 5 | UDP-glucuronosyltransferase 3A2 | Ugt3a2 | 80203;92353;99164;117089;122955 | ↑ | ↑** | ↑ |
| Q8K154;E9PZ30;D3YUX6;Q80X89;Q6PDD0 | 13;11;1;1;1 | 11;10;0;0;0 |  | Ugt2b34 | 2780;56443;87134;101500;105378;140185;141873;171670;171681;179350;202197;208200;210925 | ↓** | ↑*** | ↑*** |
| Q8R084 | 7 | 6 |  | Ugt2b1 | 2806;21896;88466;101395;141879;178682;183092 | ↓*** | ↑ | ↑* |
| Q9JJL3;Q9JJL3-2 | 5;5 | 5;5 | Solute carrier organic anion transporter family member 1B2 | Slco1b2 | 69233;97136;127731;162647;167275 | ↓*** | ↑ | ↑** |
| Q9QXZ6;E9Q0W2;Q99J94;Q91YY5 | 6;5;1;1 | 6;5;1;1 | Solute carrier organic anion transporter family member 1A1 | Slco1a1 | 46907;75464;99471;127964;154495;184705 | ↓*** | ↑** | ↑ |


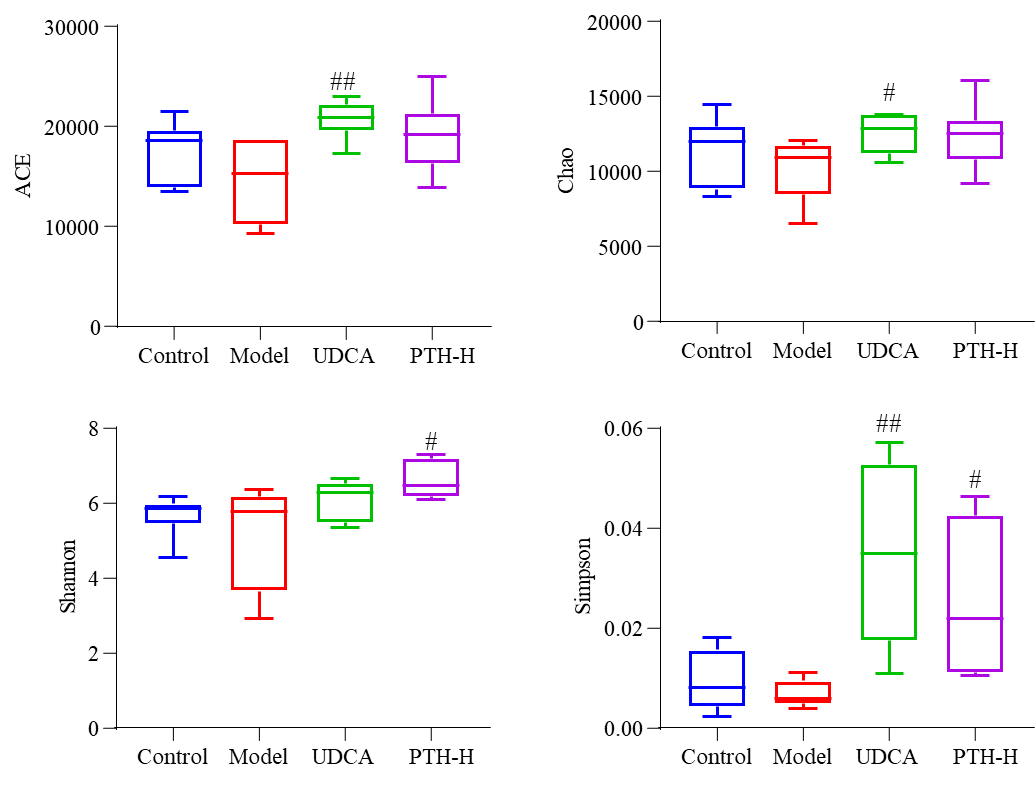


Fig S5 Comparison of α-diversity of gut microbiota including ACE, Chao1, Simpson and Shannon index between the four groups. (#*p* < 0.05 and ##*p* < 0.01, compared with the model group.)
